# Mathematical models and computational approaches in CAR-T therapeutics

**DOI:** 10.3389/fimmu.2025.1581210

**Published:** 2025-08-01

**Authors:** Guido Putignano, Samuel Ruipérez-Campillo, Zhou Yuan, José Millet, Sara Guerrero-Aspizua

**Affiliations:** ^1^ bioERGOtech, Taranto, Italy; ^2^ Department of Biosystems Science and Engineering, ETH Zurich, Basel, Switzerland; ^3^ Department of Computer Science, ETH Zurich, Zurich, Switzerland; ^4^ AI Center, ETH Zurich, Zurich, Switzerland; ^5^ Alfred E. Mann Department of Biomedical Engineering, University of Southern California, Los Angeles, CA, United States; ^6^ ITACA Institute, Universitat Politecnica de Valencia, Valencia, Spain; ^7^ Universidad Carlos III de Madrid, Departamento de Bioingeniería, Centro de Investigación Biomédica en Red de Enfermedades Raras-ISCIII, Instituto de Investigación Sanitaria Fundación Jiménez Díaz, Centro de Investigaciones Energéticas, Medioambientales y Tecnológicas, Madrid, Spain; ^8^ Centre for Biomedical Research (CIBM), University of Granada, Granada, Spain

**Keywords:** synthetic biology, biological system modeling, CAR-T cells, mathematical modeling, computational immunotherapy, therapeutic optimization, T cell engineering

## Abstract

**Background:**

The field of synthetic biology aims to engineer living organisms for specific therapeutic applications, with CAR-T cell therapy emerging as a groundbreaking approach in cancer treatment due to its potential for flexibility, specificity, predictability, and controllability. CAR-T cell therapies involve the genetic modification of T cells to target tumor-specific antigens. However, challenges persist because the limited spatio-temporal resolution in current models hinders the therapy’s safety, cost-effectiveness, and overall potential, particularly for solid tumors

**Main body:**

This manuscript explores how mathematical models and computational techniques can enhance CAR-T therapy design and predict therapeutic outcomes, focusing on critical factors such as antigen receptor functionality, treatment efficacy, and potential adverse effects. We examine CAR-T cell dynamics and the impact of antigen binding, addressing strategies to overcome antigen escape, cytokine release syndrome, and relapse.

**Conclusion:**

We propose a comprehensive framework for using these models to advance CAR-T cell therapy, bridging the gap between existing therapeutic methods and the full potential of CAR-T engineering and its clinical application.

## 1 Introduction

Cancer is a pathological condition characterized by the uncontrolled growth and metastasis of cells within the body, which evade normal cellular processes, such as programmed cell death, and disrupt immune surveillance (1, 2). Its development arises from a complex interplay of genetic predispositions, inheritance, virus exposure, and environmental factors. Despite significant research, cancer remains a major health challenge worldwide. In recent years, remarkable progress has been made in the development of cancer immunotherapies, which aim to harness the power of the immune system to fight cancer. Chimeric Antigen Receptor (CAR)-T cell therapy has emerged as a revolutionary approach in cancer immunotherapy, with seven CAR-T cell therapies approved by the Food and Drug Administration (FDA) for hematological malignancies. Despite this remarkable progress, significant challenges remain, including minimizing adverse events, achieving long-lasting and complete remissions, and extending the therapy’s efficacy to patients with solid tumors. To address these challenges, a deeper understanding of the molecular mechanisms underlying CAR-T cell response is crucial. Recently, mathematical modeling and simulation have been applied to provide systematic and quantitative analyses of CAR-T cell activity and patient responses. This review will summarize the latest research in this rapidly evolving field, categorizing the studies based on their contributions to the future of CAR-T therapy.

CAR-T cells are T lymphocytes genetically modified to express chimeric antigen receptors (CARs) that are designed to recognize and bind to specific antigens on tumor cells with high selectivity. Other types of immune cells, such as macrophages and NK cells, can also be modified to express a chimeric antigen domain. Even though some promising results involve NK cells for their lower risk (3), the most profitable area of interest involves T cells. In CAR-T cells, CARs are modular structures, consisting of an extracellular antigen-binding domain for target recognition, a hinge region for flexibility, a transmembrane domain for anchoring the receptor to the cell membrane, and intracellular signaling domains that activate the T cell upon target binding (4, 5).

The extracellular antigen-binding domain acts as an external sensor, interacting with potential target molecules of the cell surface. The domain comprises a heavy and light chain of monoclonal antibodies connected with a linker to form a single-chain variable fragment (scFv). The affinity is fundamental in determining cell function, and the scFv plays a significant role in determining the interaction with the light and heavy chains. The hinge region, also called a spacer, is a domain whose function is to enhance the flexibility of the scFv receptor head, reducing the spatial constraints between the CAR and its target antigen. Increasing the length allows the antigen binding domain to reach the antigenic determinant, also known as an epitope. The transmembrane domain connects the extracellular hinge and the antigen-binding domains with the intracellular signaling region. It could influence cell functions by increasing expression level and stability. Finally, the design of the intracellular signaling domain has undergone significant advancements over the past three decades, evolving into five distinct generations.

**Figure 1:**
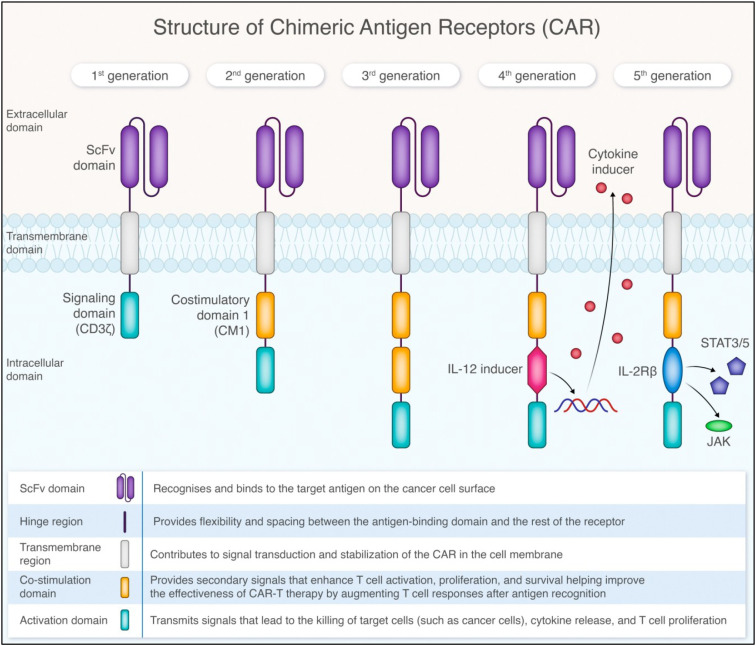
Structural comparison of five generations of Chimeric Antigen Receptor (CAR) designs. All generations contain an ScFv domain for antigen recognition and a transmembrane domain. Each subsequent generation introduces additional components: CD3ζ signaling domain (1st gen), costimulatory domain (2nd gen), multiple costimulatory domains (3rd gen), IL-12 inducer (4th gen), and IL-2Rβ with JAK/STAT3/5 signaling (5th gen). The figure depicts key functional domains and their roles in CAR-T cell activation and anti-tumor response.

In the first generation of CAR design, the intracellular domain comprised the CD3ζ cytoplasmic domain; however, the activation signal merely from CD3ζ tail was not sufficient to elicit efficient and persistent T cell function. Thus, it was quickly replaced by the second-generation CAR-T that also contains a CD28 or 4-1BB costimulatory domain in addition to mediating more potent anti-tumor activity. The third generation includes multiple costimulatory domains, such as CD28 and 4-1BB or CD28 and OX40. This design further enhances CAR-T cell efficacy, proliferation, and cytokine production and is actively used in clinics for hematological cancer treatment. The fourth generation of CAR-T cell design, also known as T cell redirected for universal cytokine-mediated killing or TRUCKs, is based on second-generation CARs with additional transgenes for cytokines secretion such as IL-2, IL-5, or IL-12. It promotes the production and secretion of the desired cytokine to promote tumor killing or to preserve a preferential CAR-T cell phenotype (i.e., memory T cell). The fifth generation of CARs is based on the second generation of CARs, containing a truncated cytoplasmic domain of cytokine receptors, such as IL-2R chain fragment, that includes a motif for binding transcription factors STAT-3/5. In this design, the antigen-induced CAR signaling effectively provides all three required signals for a complete T cell activation: antigen recognition (CD3ζ signaling), co-stimulation, and cytokine (JAK–STAT) signaling. This design further enhances CAR-T cell cytokine secretion, memory formation, reduces cytotoxicity by fine-tuned activation, and is suitable for more complex environments, such as solid tumors, by enhanced tumor infiltration and resistance to immunosuppression. **Figure 1** illustrates the structural evolution and key components of these five CAR generations, highlighting their progressive enhancement in functionality and complexity.

Upon the CAR extracellular domain engaging the specific antigen, it triggers the phosphorylation of CD3-zeta cytoplasmic domain and co-stimulatory domain(s), which further connects the CAR initiated signals to the endogenous T cell signaling pathways. The CD3-zeta mediated signal, co-stimulatory signal, together with cytokine signal induces cell activation, and proliferation, results in the clonal expansion of CAR-T in a patient. CAR engagement of the specific antigen also leads to the formation of the immunological synapse (IS) between the CAR-T cell and the antigen-expressing tumor cell. With the IS mediated cell-cell contact, CAR-T cells achieve targeted tumor killing by a synergistic mechanism of cytotoxic effector molecules delivery and Fas–FasL pathway triggering, promoted by cytokines secretion.

However, CAR-T cells present several unsolved challenges (6), such as hindrance and limited endurance in B cell malignancies, limited persistence, antigen escape, neurological complications (7), compromised immune response, hypersensitivity reactions during infusion, hematological cytopenia (8, 9), poor trafficking, limited penetration, and immunosuppressive microenvironments in solid tumors. These hurdles can be addressed by optimizing therapeutic strategy and dosage of CAR-T cell administration, exploring combination therapy, and developing next-generation CAR-T cell therapy that balances efficiency and safety by using logic-gated CAR-T cells or CAR-T cells spatiotemporally controlled by chemical or physical activation.

Achieving these improvements depends on a precise understanding of CAR-T cell dynamics and therapeutic outcomes, where computational approaches offer a timely and accessible means to provide critical insights. Mathematical models, in particular, can play a significant role in enhancing the precision, effectiveness, and durability of CAR-T therapies. By simulating complex interactions within the tumor microenvironment and predicting therapeutic responses, these models help optimize treatment regimens, reduce patient side effects, and ultimately enable higher therapeutic dosages to potentially expedite cancer eradication and reduce costs. Beyond these practical benefits, computational modeling serves as a powerful tool to accelerate discovery and translation in CAR-T cell research. It enables hypothesis generation, guides experimental design, and provides a structured framework to investigate the multiscale mechanisms underlying therapeutic success or failure. As such, these models are not merely supplementary but are increasingly integral to driving innovation and improving clinical outcomes in CAR-T therapy.

In this review, we examine studies published since 2019, selected for their relevance to the field, to present the latest advancements in computational approaches applied to CAR-T therapeutics.

## 2 Opportunities and challenges of computational modeling in CAR-T therapy

**Figure 2:**
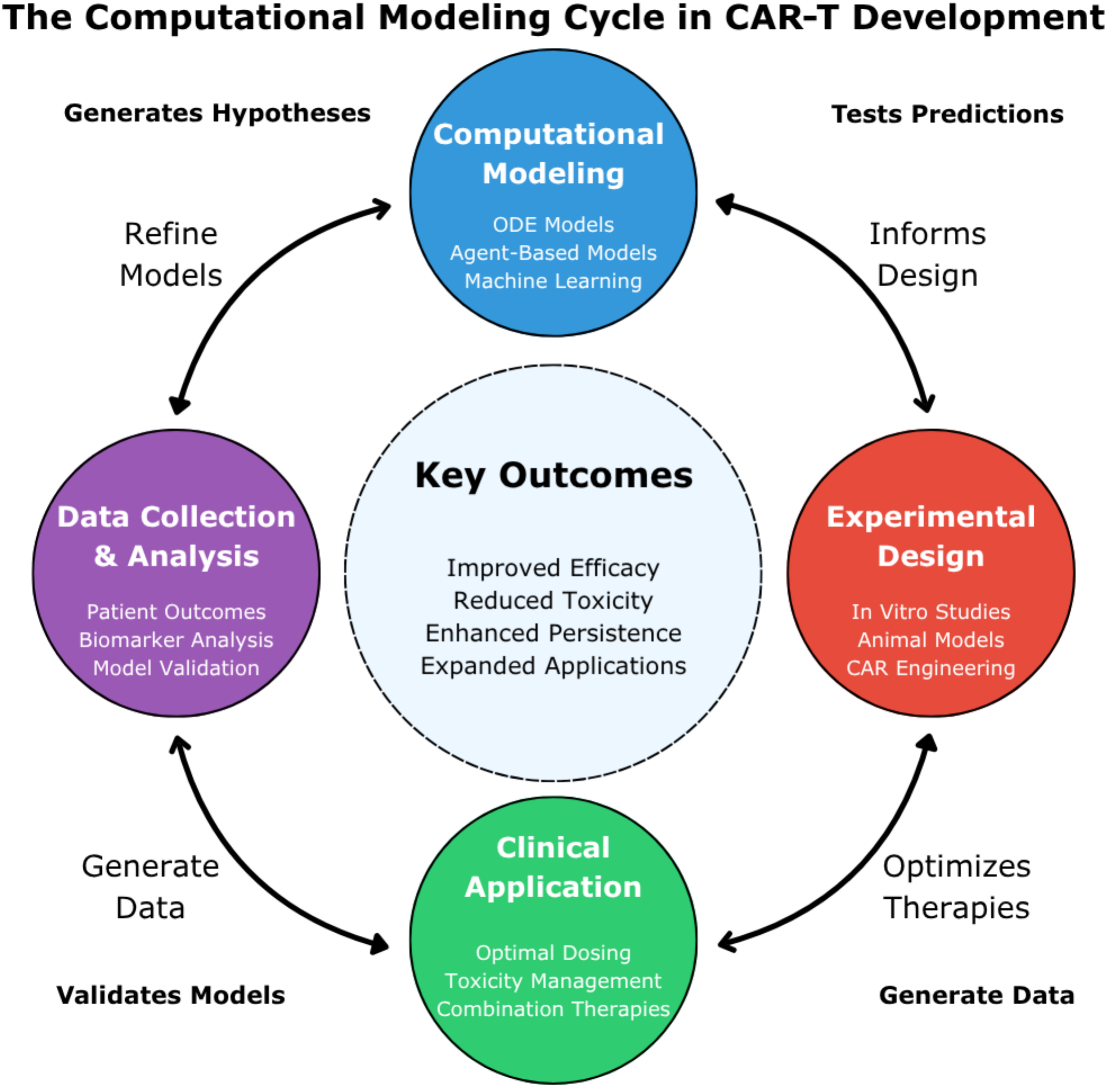
The computational modeling cycle in CAR-T development. This figure illustrates the iterative cycle of computational modeling in CAR-T cell therapy development. Four interconnected stages are represented: “Computational Modeling” (blue, top) featuring ODE models, agent-based models, and machine learning; “Experimental Design” (red, right) encompassing *in vitro* studies, animal models, and CAR engineering; “Clinical Application” (green, bottom) focusing on dosing optimization, toxicity management, and combination therapies; and “Data Collection & Analysis” (purple, left) incorporating patient outcomes and model validation. The central “Key Outcomes” highlight the ultimate goals: improved efficacy, reduced toxicity, enhanced persistence, and expanded applications. These models generate testable hypotheses about CAR-T mechanisms and therapeutic responses, which are then validated through experimental and clinical studies that continuously refine model accuracy. Bidirectional arrows indicate how each stage both informs and is informed by the others, with experimental and clinical data continuously feeding back into computational modeling for iterative hypothesis generation, testing, and model refinement.

Computational modeling has emerged as a vital component in advancing CAR-T cell therapy, offering tools to optimize experimental design, personalize treatment, and anticipate clinical outcomes. In this section, we summarize the main opportunities that modeling approaches bring to the field and discuss open challenges that future efforts must address. **Figure 2** provides an overview of the computational modeling cycle in CAR-T therapy, highlighting its iterative nature and the key stages where modeling contributes to development and clinical success.

### 2.1 Guiding experimental design and reducing development costs

Computational models significantly reduce the experimental burden of CAR-T cell development through several complementary mechanisms. Parameter space exploration represents a primary advantage, as evidenced by Finley et al. (10), whose models efficiently examined vast arrays of CAR design options that would have been prohibitively expensive and time-consuming to test experimentally. Their work on NFκB signaling pathways identified key kinetic parameters affecting CAR-T cell response time, thereby directing experimental focus to specific modifications of the 4-1BB co-stimulatory domain. Furthermore, computational approaches facilitate hypothesis prioritization, as demonstrated by Greenman et al. (11), whose models identified previously underappreciated factors such as receptor downmodulation that significantly influence CAR-T cell function. By highlighting these critical mechanisms, such approaches enable researchers to prioritize specific hypotheses for experimental testing. Resource optimization constitutes another vital benefit, particularly since CAR-T cell manufacturing remains expensive and labor-intensive. Models developed by Barros et al. (12) provide insights into optimizing cell expansion protocols, potentially reducing manufacturing costs and increasing therapy accessibility across diverse patient populations.

The integration of computational approaches with manufacturing optimization represents a critical frontier for improving CAR-T cell therapy outcomes. Colina et al. (13) provided a comprehensive framework highlighting how computational models can inform manufacturing decisions across the entire production pipeline, from T cell activation and expansion protocols to construct selection and delivery methods. Their review demonstrates how mathematical models can guide key manufacturing variables including media composition, isolation and depletion of specific cell subsets, activation strategies, and construct delivery methods. This manufacturing-focused computational approach complements traditional pharmacokinetic/pharmacodynamic modeling by addressing the upstream factors that determine the quality and functionality of the final CAR-T cell product before infusion. The authors emphasize that computational models can help optimize manufacturing conditions to favor less differentiated, more persistent CAR-T cell phenotypes, which correlates with improved clinical outcomes (13).

### 2.2 Improving patient outcomes through predictive modeling

Computational approaches directly contribute to improved patient outcomes through several interconnected mechanisms. Personalized dosing strategies represent a significant advancement, as demonstrated by Valle et al. (14) and Levin et al. (15), whose mathematical modeling work determines optimal dosing regimens tailored to individual patients, potentially reducing toxicity while maintaining efficacy. Their models suggest that lower, precisely calculated doses may achieve complete responses while minimizing side effects—a finding that challenges the conventional “more is better” approach prevalent in many therapeutic contexts. Side effect prediction and management constitute another critical contribution, exemplified by Zhang et al. (16), who developed models that predict cytokine release syndrome (CRS) severity and identify optimal timing for interventions such as tocilizumab administration. These models indicate that preemptive tocilizumab could reduce CRS severity by 25% without compromising therapeutic efficacy, a prediction subsequently validated in clinical trials. Additionally, computational approaches enable relapse prediction, as evidenced by Liu et al. (17), who created models that can predict relapse probabilities based on early response data, potentially allowing for preemptive intervention before clinical deterioration becomes evident. Their work identifies specific biomarkers that could be monitored to predict treatment failure, enabling earlier clinical interventions. Furthermore, combination therapy optimization has advanced through studies by Adhikarla et al. (18) and Mahasa et al. (19), who employ mathematical modeling to identify synergistic treatment combinations and optimal sequencing of CAR-T cells with other therapies. Their research demonstrates that administering CAR-T cells before other treatments can significantly increase progression-free survival, providing actionable insights for clinical protocol development.

### 2.3 Critical unanswered challenges for future modeling

Despite significant progress, several critical challenges remain that future computational models must address. Solid tumor penetration and efficacy represent a primary concern, as current models inadequately capture the complex spatial dynamics of solid tumors. Future models must incorporate multiple factors, including physical barriers to T cell infiltration, immunosuppressive tumor microenvironments, heterogeneous antigen expression across tumor regions, and oxygen and nutrient gradients affecting CAR-T cell function. These elements collectively influence therapeutic efficacy and require sophisticated spatial modeling approaches to accurately simulate the tumor-immune cell interface. Long-term persistence and memory formation constitute another challenge requiring advanced computational frameworks to better understand and optimize the transition of CAR-T cells from effector to memory phenotypes, which is critical for durable responses. These models should predict factors governing memory cell formation, persistence duration under various conditions, and strategies to enhance memory without compromising initial efficacy—a balance critical for sustained therapeutic outcomes. As CAR designs become increasingly complex with multi-antigen targeting systems such as logic-gated CARs and dual-targeting systems, sophisticated computational frameworks must be developed to predict their behavior in heterogeneous tumor environments and optimize their design parameters. Real-time adaptation represents another frontier, as future models should work toward real-time integration of patient data to allow dynamic adjustment of treatment parameters throughout therapy, creating truly adaptive treatment protocols that respond to evolving patient conditions. Finally, biomarker identification remains crucial, as computational approaches could identify novel biomarkers that predict response and toxicity, enabling better patient selection and monitoring. These models should integrate multi-omic data with clinical parameters to develop comprehensive predictive frameworks that enhance clinical decision-making and treatment personalization.

### 2.4 Hypothesis generation and testing through computational frameworks

A fundamental strength of computational modeling in CAR-T therapy lies in its ability to generate testable hypotheses about cellular mechanisms and therapeutic responses. Mathematical models can propose non-intuitive relationships between CAR design parameters, manufacturing conditions, and clinical outcomes that would not be apparent through experimental observation alone. For example, models suggesting that lower CAR-T cell doses might achieve superior killing efficiency per cell while increasing exhaustion rates generate specific hypotheses about dose-response relationships that can be experimentally validated.

Computational frameworks enable systematic hypothesis testing by allowing researchers to simulate “what-if” scenarios under controlled conditions before committing to expensive experimental validation. Models can predict how specific perturbations—such as modifying costimulatory domains, altering manufacturing protocols, or adjusting dosing regimens—will affect therapeutic outcomes. These predictions generate explicit hypotheses that guide targeted experimental design and clinical trial planning, creating an iterative cycle where computational predictions inform experimental work, and experimental results refine model accuracy and generate new hypotheses for testing.

## 3 Modeling in describing CAR-T cell response

**Figure 3:**
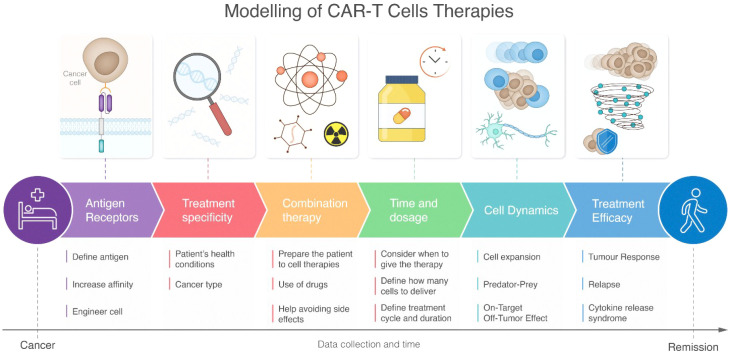
Schematic representation of CAR-T cell therapy modeling pipeline from disease state to recovery. The workflow progresses through six interconnected stages: antigen receptors, treatment specificity, combination therapy, time and dosage, cell dynamics, and treatment efficacy. Each stage includes specific modeling considerations depicted by icons and detailed subpoints below. The pathway flows from initial patient condition to healthy outcome, with a timeline emphasizing continuous data collection throughout the treatment process. The figure highlights both molecular-level considerations (such as antigen receptor engineering) and systemic responses (like cytokine release syndrome), demonstrating the comprehensive nature of CAR-T therapy modeling.

This chapter provides a comprehensive overview of research utilizing mathematical modeling to investigate the CAR-T cell response. As depicted in **Figure 3**, the modeling pipeline spans from cancer to remission, encompassing key aspects such as antigen receptor design, treatment specificity, and cell dynamics. The first sub-section discusses how modeling is employed to characterize CAR-T cell signaling and function in response to various parameters. These parameters include the affinity of CAR binding to tumor antigen, the density and heterogeneity of presented tumor antigen, variations in CAR designs (including co-stimulatory domains), and different CAR-T cell to tumor cell ratios. The second sub-section explores how modeling can be used to predict the *in vivo* response of CAR-T cells after encountering tumor cells, including its proliferation, expansion, trafficking, killing, pharmacological response, and potential side effects.

### 3.1 Dissect cellular response of CAR-T cells

#### 3.1.1 Antigen expression predicts treatment efficacy

Fischel and colleagues (20) investigated the minimum percentage of antigen expression needed in a tumor for effective treatment. Their findings suggest a negative correlation between the percentage of antigen-presenting tumor cells and tumor growth. Moreover, the study highlights the potential challenge of CAR-T cells reaching their targets due to a “shield” of antigen-negative cells surrounding antigen-presenting cells. The example used is Triple-Negative Breast Cancer, which lacks the receptors typically targeted by therapies (estrogen, progesterone, and human epidermal growth factor receptors).

Tumors with a high number of antigen-presenting cells showed significant reduction. However, identifying a universal antigen across all cancer types remains a challenge. The study suggests that tumor size reduction might still be achievable even if more than 50% of the tumor is non-antigen-presenting. Furthermore, the antigen composition of a tumor appears to influence the number of CAR-T cells needed for therapeutic efficacy. Antigen receptors can be especially important when matched with full spatiotemporal control to sense specific ligands and control gene expression such as in the case of Synthetic juxtacrine receptors (SJRs) (21).

#### 3.1.2 Downstream signaling events

The mathematical modeling and computational approaches were further utilized to examine CAR-T cells signaling. Naghizadeh et al. (22) employed a machine learning-based detection and segmentation method to assess CAR-T cell immunological synapses in patients. Their approach specifically utilized instance segmentation, as cells display clustering, irregular shapes, and occlusion. By leveraging these techniques, the authors were able to develop new procedures for studying the immunological synapses between T cells and antigen-presenting cells.

The potency of downstream signaling events triggered by antigen binding depends on the co-stimulatory domain within the structure of the CAR. CAR-T cells engineered with 4-1BB or CD28 co-stimulatory domains have demonstrated significant anti-tumor activity. Previous studies have shown that CD28-based CAR elicits stronger and more sustained signaling compared to 4-1BB counterparts, which exhibit milder activation but potentially longer persistence. However, traditional experimental comparison across various tumor models to assess their performance and signaling response is laborious and time-consuming. Using ordinary differential equation-based mathematical models and Monte Carlo simulations, Finley et al. (10, 14, 23) elucidated how CAR co-stimulatory domains influence cell activation in the presence of biological variability. Their work quantitatively described the NFκB signaling pathway activated by CD19scFv-4-1BB and proposed specific manipulations to refine the response of these cells at physiological antigen. Additionally, they also demonstrated a faster and more consistent population response, mediated by CD28-based CARs. Their findings identified kinetic parameters impacting cell response time and proposed strategies to prolong response times in heterogeneous CAR-T cell populations by enhancing lymphocyte-specific protein tyrosine kinase activity.

Reiter et al. (11) highlighted the importance of integrating mathematical and experimental approaches during model development. Their work employed a phenotypic model comprised of ordinary differential equations to describe the interplay between biophysical parameters of CAR binding (affinity, avidity, and antigen density) and CAR-T cell activity. By incorporating experimentally derived parameters and analyzing receptor downmodulation and intracellular signaling processes, they revealed that receptor downmodulation is a previously underappreciated factor influencing CAR-T cell function.

Shah et al. (24) took a different computational approach by developing a coarse-grained logic-based Boolean model to simulate the evolution of signaling signatures during CAR-T cell and tumor cell interactions. Using probabilistic Boolean networks (PBNs), their model incorporated major activation pathways (CAR, MAPK, PI3K-AKT-MTOR1, calcium signaling), cytokine signaling (IL2), cytotoxic pathways, and inhibitory signaling (PD1 and CTLA4) with relevant crosstalks and feedback mechanisms. The model employed three readout nodes capturing CAR-T cell function (CFUNC), CAR-T cell inhibition (CINHIB), and tumor apoptosis (TAPOP) to track signaling signature trajectories. Their simulations revealed that while activation of the CAR receptor alone leads to moderate functional and cytotoxic signaling, addition of IL2 increases functional signaling, whereas inhibitory pathways through PDL1L2 and CD8086 markedly decrease functional signatures. Importantly, their model predicted that significant improvements to baseline CAR-T cell function through intracellular network perturbations may be intrinsically limited, suggesting that optimization strategies should focus on supporting sustained low-level activation rather than maximum activation (24).

### 3.2 Predict *in vivo* response of CAR-T cell

In order to fully exploit the potential of engineered CAR-T cells *in vivo*, it is crucial to consider their behavior and interactions within the intricate biological environment. This section presents a comprehensive overview of the mathematical models that have been created to assist in comprehending and optimizing CAR-T cell therapy.

#### 3.2.1 CAR-T cell dynamics

In CAR-T cell therapy, after cell infusion, there is a well-defined sequence of phases: distribution, expansion, and contraction (25). Upon infusion, CAR-T cells enter the bloodstream and distribute to various tissues throughout the body. This initial phase is characterized by a transient decrease in circulating CAR-T cell density. Once reaching the tumor site, the CAR-T cells undergo a robust expansion phase, proliferating several orders of magnitude to reach a peak density (Cmax) typically within 2–3 weeks post-infusion (26, 27). This expansion is driven by the recognition and engagement of tumor-associated antigens (TAAs) by the CAR-T cells. CAR-T cells undergo contraction due to activation-induced cell death (AICD) (5). This process is thought to be a self-regulatory mechanism to prevent excessive immune activation and potential toxicities. A fraction of CAR-T cells adopts memory phenotypes and persists for months to years to mediate continuing antitumor activity. These memory cells are crucial for preventing tumor relapse.

Paixao et al. (28) investigated the impact of antigen specificity and cellular dynamics within the tumor microenvironment on CAR-T cell expansion. The study focused on patients with hematological malignancies, suggesting that analyzing the concentration of non-exhausted CAR-T cells could potentially predict future treatment responses.

Researchers often model distinct phases of CAR-T therapy independently to gain specific insights. Liu et al. (29) observed that CAR-T responders have a higher expansion capacity, greater proliferation, and lower contraction rates than non-responders. CAR-T cells proliferate at a relatively higher rate in hematologic malignancies than in solid tumors, a finding applicable to both solid and liquid tumors. Hematologic malignancies (including ALL/CLL/MM) display greater proliferation than solid tumors and lymphomas. This difference can be attributed to the challenges CAR-T cells face in penetrating solid tumors and recognizing antigens. Additionally, the study revealed age-related disparities in patient responses. Pediatric ALL patients displayed lower proliferation rates, contraction, and memory cell death, while exhibiting higher memory differentiation compared to adults. These findings underscore the importance of incorporating patient-specific factors into CAR-T therapy models.

External factors such as medication can also influence the dynamics of CAR-T cells. For instance, dexamethasone, a steroid medication used to reduce inflammation and suppress the immune system, can affect CAR-T cells. Brummer et al. (30), reported that high doses of dexamethasone destabilize the *in vitro* coexistence between tumor cells and CAR-T cells. This drug can decrease the activity of CAR-T cells and lead to CAR-T exhaustion, which ultimately promotes the growth of tumor cells. Another important consideration when developing new therapeutics is the threshold-dependent tolerance of CAR-T cells. Brummer et al. (30) noted that CAR-T cells effectively eliminate cancer cells up to a certain tolerability threshold; beyond this threshold, however, high doses of dexamethasone impair their efficacy.

#### 3.2.2 CAR-T cell expansion

Enhancing CAR-T cell expansion, both *in vitro* and *in vivo*, can increase the number of cells available for patient infusion. Carvalho Barros et al. (12) focused on *in vivo* cell expansion, in order to reduce potential treatment offsets. They built upon a previous paper (31) which explained the possibility of studying CAR-T cells *in vivo* by directly correlating the number of tumor cells with photon emission, even though the exact number cannot be determined), they developed a model that did not account for natural tumor cell death or immune evasion. Deterministic models, mathematical frameworks in which system parameters uniquely determine the outcome with no randomness involved, have been used to understand CAR-T cell behavior. In these models, the same initial conditions always produce identical results, making them computationally efficient but potentially less reliable when modeling systems with low cell numbers where random fluctuations can have significant effects.

To address this limitation, Kimmel et al. (32) employed a hybrid (deterministic-stochastic) model for a more detailed description of cell dynamics. This approach combines deterministic equations for larger cell populations with stochastic elements that account for random variations when cell numbers are small, thus leveraging the computational efficiency of deterministic models with the biological realism of stochastic approaches. CAR-T cells typically reach a peak followed by decay, with fitting parameters informed by previous data (33, 34). These authors also distinguished the dynamics of CAR-T cells from normal T cells, noting the latter follow a stable first-order response. Additionally, in a prior study (35), they investigated how the system evolves under different conditions by comparing progression-free survival based on variations in parameters such as tumor growth rate, initial tumor size, memory CAR-T cell fraction, and T cell density at CAR injection.

#### 3.2.3 CAR-T cell killing

Agent-based modeling, particularly leveraging predator-prey dynamics, mathematical frameworks originally developed in ecology to describe interactions between hunters and their prey, has been instrumental in exploring CAR-T cell killing kinetics. Predator-prey mathematical models play a crucial role in understanding the interactions between CAR-T and other cells at the tissue level. Agent-based modeling, particularly Predator-prey dynamics have been used to explore CAR-T cell killing kinetics. Predator-prey dynamics models represent the relationship between two populations where one (predator) relies on the other (prey) for survival.

Agent-based modeling approaches have provided valuable insights into CAR-T cell killing dynamics. Luque et al. (36) developed an agent-based model to study heterogeneous tumor-derived organoid response to CAR-T cell therapy, demonstrating that increasing CAR-T cell dosage does not necessarily improve killing efficiency. Their simulations revealed an emergent ‘shield-like’ structure formed by cells with low antigen expression that protected cells with high antigen expression, highlighting how spatial organization influences therapeutic outcomes.

Brummer et al. (37) pioneered the application of Sparse Identification of Nonlinear Dynamics (SINDy) algorithms to biological systems. SINDy is a computational method that automatically discovers the underlying mathematical equations from experimental data by identifying the minimal set of terms needed to accurately represent the system’s behavior, essentially reverse-engineering the rules governing cellular interactions from observed patterns. In other words, SINDy identifies the underlying equations governing a system from a set of observed data (38), which modeled cancer cell growth with logistic functions and considered multiple factors influencing CAR-T cells, such as rates of proliferation, exhaustion, persistence, and target cell killing. They found an inverse relationship between CAR-T cell dose and killing rate, but a direct relationship with the rate of exhaustion and proliferation. Lower doses of CAR-T cells would kill more cancer cells for each T-cell, but they would also lead to faster exhaustion and subsequent tumor regrowth.

Based on the matrix parameters of the model, it is possible to obtain specific results from the system. The results suggest that it is crucial to consider the proliferation and exhaustion of CAR-T cells apart from how many cancer cells can be eliminated. The CARRGO model (Chimeric Antigen Receptor T cell treatment Response in GliOma) is highly accurate for *in vivo* or *in vitro* studies of humans and has the potential to be used in real-life scenarios. One key limitation of Predator-Prey models is their inability to capture the full dynamic range of CAR-T cell behavior. They cannot fully represent the possibility of a CAR-T cell showing its potential to change from an effector CAR-T cell into a memory cell and then back to a CAR-T effector cell after identifying an antigen. Barros et al. (39) addressed this limitation by understanding how a tumor can inhibit immune cells. In their model, it is possible to observe that the fast decay of CAR-T cell doses may prevent CAR-T cells from becoming memory cells. The model was derived and modified from a prior empirical model of the immune response to bacterial or viral infections that can demonstrate how different problems that follow similar dynamics can potentially be described with few differences. The model described did not take into consideration the toxicity effect of CAR-T cell immunotherapy. However, they introduced the CARTmath platform for studying tumor responses to CAR-T cell immunotherapy in immunodeficient mouse models of hematological cancers.

Mathematical approaches to modeling CAR-T cell pharmacology have been further advanced by Kirouac et al. (40), who developed quantitative frameworks to analyze the complex relationships between product characteristics, patient physiology, and clinical outcomes. Their study demonstrated that CAR-T pharmacokinetics can be separated into distinct phases including biodistribution, expansion, contraction, and persistence. Notably, their modeling revealed that both maximal cell expansion (Cmax) and immunophenotype of circulating CAR-Ts following expansion are predictive of patient response, with patients showing robust tumor responses more likely to have pharmacokinetic profiles in the top quartile of the distribution. Their work highlights how mathematical models can characterize the high interpatient variability in CAR-T cell exposure and efficacy, which significantly exceeds the variability typically observed with small molecules or biologics.

##### 3.2.3.1 Mathematical formulation of predator-prey dynamics in CAR-T cell therapy

To illustrate the mathematical approaches used to model CAR-T cell and tumor interactions, we present the CARRGO (Chimeric Antigen Receptor T cell treatment Response in GliOma) model developed by Sahoo et al. (38). This model utilizes a predator-prey framework described by the following system of ordinary differential equations:


$$\frac{dX}{dt}=\rho X\left(1-\frac{X}{K}\right)-k_{1}XY$$

$$\frac{dY}{dt}=k_{2}XY-\theta Y$$

Where X represents cancer cell density, Y is CAR-T cell density, ρ is the net growth rate of cancer cells (day^−1^), K is the cancer cell carrying capacity, k_1_ is the killing rate of CAR-T cells (day^−1^ cell^−1^), k_2_ is the net rate of proliferation including exhaustion of CAR-T cells when encountered by a cancer cell (day ^−1^ cell^−1^), and θ is the death rate of CAR-T cells (day ^−1^).

The first equation represents cancer cell dynamics, with logistic growth $\rho X\left(1-\frac{X}{k}\right)$ and cell death $k_{1}XY$ due to CAR-T cell killing. The second equation describes CAR-T cell dynamics, where $k_{2}XY$ represents either proliferation (if $k_{2}$ *>0*) or exhaustion (if $k_{2}$ <*0*) of CAR-T cells upon encountering cancer cells, and *θY* accounts for natural CAR-T cell death.

Through non-dimensionalization, the system can be rewritten as:


$$\frac{dx}{dt}=x\left(1-x\right)-xy$$

$$\frac{dy}{dt}=Bxy-Ay$$

With dimensionless parameters *A =* $\frac{\rho }{\theta }$ and B = $\frac{k_{2}K}{\rho }$ .

Dynamical systems analysis reveals three distinct therapeutic outcomes based on parameter values:

- Successful CAR-T cell treatment (*A=0, B>0*): Cancer cells are eliminated with some remaining CAR-T cells. This occurs when CAR-T cell persistence is high and proliferation exceeds exhaustion.
- CAR-T cell treatment failure (*A=0, B<0*): Cancer cells grow to carrying capacity and CAR-T cells are eliminated, occurring when exhaustion dominates proliferation.
- Pseudo-failure/pseudo-response (*A>0, B>0*): Cancer and CAR-T cells coexist in oscillatory patterns, potentially explaining the clinical phenomenon of pseudo-progression.

The CARRGO model demonstrates that the balance between proliferation and exhaustion (*k2*) is more critical for treatment success than the killing rate (*k1)*. Experimental validation showed that CAR-T cell dose inversely correlates with killing rate and directly correlates with proliferation/exhaustion rate, suggesting that at lower doses, individual T cells kill more cancer cells but become more exhausted compared to higher doses.

This mathematical framework provides quantitative insights into key factors influencing CAR-T therapy outcomes and highlights the importance of considering nonlinear dynamics when designing and optimizing treatment protocols.

**Biological context and CAR design implications**

The CARRGO model parameters directly relate to CAR construct components shown in **Figure 1**. The killing rate (k_1_) is influenced by the CAR’s cytotoxic signaling domains and scFv binding affinity, while the proliferation/exhaustion balance (k_2_) depends on costimulatory domain selection (CD28 vs. 4-1BB) and intracellular signaling strength. This model can inform CAR design by predicting optimal binding affinities that maximize the k_2_/k_1_ ratio for sustained therapeutic response. For therapeutic strategies, the model suggests that optimizing CAR persistence (minimizing negative k_2_) may be more critical than maximizing immediate killing efficiency, guiding the selection of costimulatory domains that favor memory formation over rapid effector function.

Within the iterative framework of **Figure 2**, this model generates hypotheses about optimal CAR designs that can be tested through experimental validation of different construct combinations, with clinical data refining parameter estimates for patient-specific modeling.

##### 3.2.3.2 T cell competition and stochastic extinction model

Another important mathematical approach is presented by Kimmel et al. (32), who developed a model that incorporates competition between normal T cells and CAR-T cells, while treating tumor elimination as a stochastic process:


$$\frac{dN}{dt}=-r_{N}N\ln\left(\frac{N+C}{K_{N}}\right)$$

$$\frac{dC}{dt}\;=-r_{C}\;\left(T\right)C\ln\left(\;\frac{N+C}{K_{C}}\;\right)$$

$$\frac{dB}{dt}\;=r_{B}B-\frac{\gamma_{B}BC}{K_{B}+C}$$

Where N represents normal T cell density, C is CAR-T cell density, B is tumor cell density, T = N + C is the total lymphocyte count, *K_N_* and *K_C_* are the respective carrying capacities, and *γ_B_* is the tumor-killing rate. The model uses Gompertz growth for immune reconstitution rather than logistic growth.

The CAR-T growth rate function *r_C_* (T) includes feedback from total lymphocyte count:


$$r_{C}\left(T\right)=\rho_{C}+b\frac{{\left(T-K_{N}\right)}^{2}}{a\cdot T^{2}+{\left(T-K_{N}\right)}^{2}}$$

A key innovation in this model is its treatment of tumor eradication as a stochastic rather than deterministic process. When tumor burden falls below a threshold (approximately 100 cells), the model switches from deterministic to stochastic simulation, allowing for calculation of extinction probabilities.

This approach revealed that:

- CAR-T cells initially expand faster but are eventually outcompeted by normal T cells.
- Cure (tumor extinction) typically occurs early (days 20-80), while progression occurs much later (days 200-500).
- Lymphodepletion before CAR-T infusion is critical for creating space for CAR-T expansion.

##### 3.2.3.3 Comparative perspectives on mathematical approaches

The mathematical models described above offer complementary insights into CAR-T cell therapy dynamics. While the CARRGO model focuses on the direct interaction between tumor and CAR-T cells and emphasizes the balance between killing efficiency and exhaustion, the Kimmel model incorporates competition with normal T cells and stochastic extinction events, helping explain variability in clinical responses.

These different mathematical formulations highlight how various modeling approaches can capture distinct aspects of the complex biological phenomena in CAR-T therapy. The CARRGO model suggests optimizing CAR-T dose based on tumor antigen expression, while the Kimmel model emphasizes the importance of effective lymphodepletion and predicts the timing of treatment outcomes.

**Biological context and clinical applications**

The Kimmel model parameters reflect real CAR-T manufacturing and clinical variables. The lymphocyte carrying capacities (K_N_, K_C_) relate to patient lymphocyte counts post-lymphodepletion, while the competition dynamics inform optimal CAR-T dosing relative to endogenous T cell recovery. The stochastic extinction threshold (~100 cells) provides a biological basis for minimum effective doses. This model can guide therapeutic strategies by predicting optimal lymphodepletion intensity and CAR-T infusion timing to maximize the therapeutic window before normal T cell recovery.

For CAR design applications, the model suggests that constructs favoring rapid initial expansion (high ρ_C_) may be more effective than those optimized for long-term persistence when administered with appropriate lymphodepletion. Within **Figure 2**’s framework, this model exemplifies how computational predictions about optimal dosing and timing can be validated through clinical trials, with patient pharmacokinetic data refining model parameters for precision dosing strategies.

#### 3.2.4 Tumor response

The term “tumor response” refers to the changes observed in a cancer following a therapeutic intervention. Responses are categorized as a complete response, partial response, stable disease, or progressive disease based on tumor size changes. Rodrigues et al. (41) developed a mathematical model that characterizes tumor elimination, equilibrium, and escape in solid tumors. Their model suggests that complete cancer cell elimination occurs in only 5% of cases, with 18% reaching equilibrium and 77% experiencing escape. Factors influencing complete elimination include tumor proliferation rate, CAR-T cell inhibition by the tumor microenvironment, and CAR-T cell proliferation and death rates. The model was based on data from HDLM-2 and Raji cell lines (42, 43).

The “stem cell hypothesis” proposes that cancer stem cells play a crucial role in tumor development and treatment resistance. Mathematical models that incorporate the effect of cancer stem cells in the tumor microenvironment are needed to understand the potential for complete response to treatment. Swanson et al. (44) developed a strategy for targeting tumor stem cells in solid tumors by infusing trained CAR-T cells. Their work highlights the role of transforming growth factor TGF-β (B) as a defensive mechanism employed by tumor cells to avoid effectors cells attack. However, TGF-β inhibitor can enhance effector cells efficacy by rendering them insensitive to the effect of TGF-β.

#### 3.2.5 Relapse

Relapse, defined as the return of cancer after treatment, can occur either locally (original site), or distantly (new location). Due to its high frequency, mathematical and computational simulations can aid in designing new treatments that aim to predict when relapse may occur. Liu et al. (17) developed a computational approach to predict long-term treatment effects based on early-stage clinical data. Using simulated clinical data allows for comparison with real-world outcomes. The authors considered the presence of the CD19 protein, found on the surface of B-cells, which is a common target for CAR-T cell therapies. Tumor cells that lose CD19 expression can evade CAR-T cell recognition, rendering the therapy less effective. Their model predicted that CAR-T reinjection might not be highly beneficial, but CAR-T cell expansion within the patient could be possible due to the initial presence of a large number of targets. However, complete tumor eradication might not be achieved due to antigen escape (tumor cell loss of target protein) or CAR-T cell fratricide (self-destruction).

Since CAR-T therapy is relatively new to the pharmaceutical industry, multiple generations of chimeric antigen receptors differ based on their efficacy and safety. Pérez-García et al. (45) underlined how the therapy design impacts predicted outcomes. Personalized medicine aims to develop patient-specific models, instead of generalized ones that may not be universally applicable.

Martínez-Rubio et al. (46) developed a dynamic model combining B cells and effector and memory CAR-T cells. This model allows for *in silico* testing of CAR-T cell dose effectiveness, helping determine optimal treatment strategies.

Bone marrow may play an important role in relapse, as it produces a constant number of B cells, but it is difficult to implement the model *in vivo*. However, as most clinical data are based on peripheral blood samples, the significance of B cells can still be assessed.

Moreover, another cause of relapse can be the presence of alternative isoforms in cancer cells, so the immune system does not detect them. These antigen-negative relapses tend to be a huge problem to consider when developing new therapeutics to avoid poor CAR-T cell cytotoxicity or persistence. For this reason, Santurio et al. developed a PDE model to define different dynamics of patients depending on the therapeutic response (47).

Overall, mathematical and computational simulations are valuable tools for designing new treatments and predicting relapse in CAR-T therapy. The development of personalized medicine and dynamic models that capture the complexity of the immune system will be crucial for improving treatment efficacy.

### 3.3 Side effects

**Figure 4:**
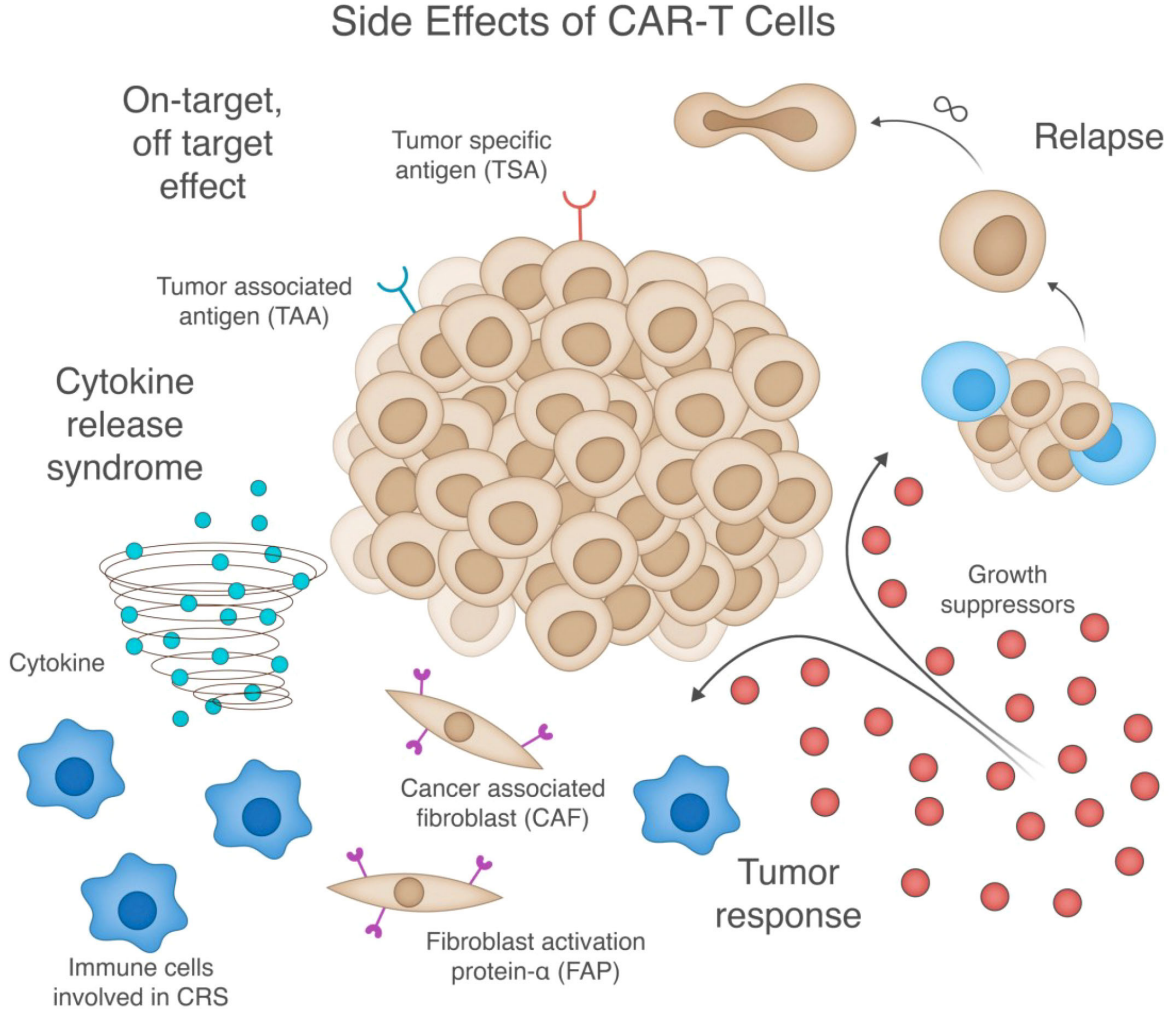
Comprehensive illustration of key side effects and challenges in CAR-T cell therapy. The central tumor mass (middle) expresses both tumor-specific antigens (TSA) and tumor-associated antigens (TAA), and is surrounded by four major therapeutic challenges: (1) Cytokine release syndrome (bottom left) - depicted by the cytokine storm and activated immune cells that contribute to systemic inflammation; (2) On-target/off-target effects (top left) - where CAR-T cells recognize antigens on non-tumor cells; (3) Tumor response mechanisms (bottom right) - including growth suppressors and resistance pathways; and (4) Potential relapse (top right) - showing tumor cells that have escaped immune surveillance. While these challenges can occur independently, they often interact—for example, tumor resistance can lead to relapse, while excessive CAR-T activation can cause both cytokine release syndrome and off-target toxicity. The figure also highlights cancer-associated fibroblasts (CAF) and their expression of fibroblast activation protein-α (FAP) in the tumor microenvironment, which can serve as both potential targets and obstacles for CAR-T therapy by modulating the tumor microenvironment and influencing CAR-T cell infiltration and function.

A significant challenge in CAR-T cell therapy is the management of adverse side effects. A range of them can occur, including on-target, off-tumor effects and systemic inflammatory responses. **Figure 4** depicts these key challenges, including cytokine release syndrome, on-target/off-target effects, tumor response mechanisms, and factors contributing to potential relapse. Understanding the underlying mechanisms of these side effects is crucial for developing strategies to mitigate them.

#### 3.3.1 On-target off-target effect

On-Target, Off-Tumor (OT, OT) responses, also known as “collateral damage,” occur when a therapeutic intervention, such as a drug or treatment, targets a specific molecule or pathway in cancer cells but unintentionally affects normal, non-cancerous cells. This can lead to toxicity or other adverse effects for the patient. For instance, CAR-T cell therapies targeting CD19, a protein expressed on both cancer and B cells can deplete B cells resulting in infections or autoimmune disorders.

Santurio et al. (48) explored this phenomenon in glioblastoma, considering the tumor population, CAR-T cells, neurons, and Glial cells expressing the antigen. Assuming 90% of glial cells lack the antigen, the study highlighted the potential for lethal central nervous system toxicity (49), due to OT, OT effects in solid tumors, despite a positive antitumor response. A higher CAR-T cell load leads to a faster and more pronounced peak, while glioblastoma cells decrease faster.

The simulation also revealed a threshold for the number of tumor cells, suggesting that quantifying CAR-T cells for effective therapy is crucial. While this work assumes direct tumor site injection, the ability of CAR-T cells to penetrate the brain-blood remains a significant challenge of current therapies.

León-Triana and colleagues (50) described a similar OT, OT interaction between CAR-T and B cells, inspired by Kuznetsov’s model (51). They investigated controlling tumor growth with single and double CAR-T therapy, concluding that dual therapy is more efficient.

The potential interaction with CD19 in the OT, OT scenario may lead to early CAR-T cell amplification. As solid tumors exhibit high immunosuppressive capabilities, an *in-silico* study proposed targeting two antigens, CD19 and a tumor-associated antigen, to produce substantial CAR-T cell numbers and overcome suppression.

### 3.4 Cytokine release syndrome

Cytokine Release Syndrome (CRS) is a common adverse effect following CAR-T cell therapies. It results from excessive cytokine release into the bloodstream, leading to various side effects such as fever, fatigue, and low blood pressure, potentially causing organ damage or death. Previous studies have shown that excessive cytokine release typically occurs within the first 14 days after CAR-T cell infusion.

Zhang et al. (16) aimed to identify factors reducing CRS probability by understanding cellular interactions. They demonstrated that tocilizumab, a medication used for rheumatoid arthritis, can lower CRS severity by 25% by blocking the interleukin-6 receptor when administered from the day of CAR-T cell infusion. Clinical trials confirmed the validity of this approach (52). Additionally, slower CAR-T cell infusion rates correlate with milder CRS symptoms. However, reducing the number of injected CAR-T cells may compromise their anti-cancer efficacy.

Corticosteroids are commonly used to reduce inflammation and suppress the immune system, potentially slowing down the immune response by decreasing cytokine production. Stein et al. (53) conducted a model-based analysis to characterize the kinetics of tisagenlecleucel therapy, a CAR-T therapy for leukemia, focusing on potential comedications for CRS. Patients with CRS have shown benefits from tocilizumab and corticosteroid treatment.

Furthermore, small molecules may potentially enhance cell therapies. However, due to the longer half-life of CAR-T cells compared to small molecules, standard pharmacokinetic parameters cannot predict their clearance. Understanding the optimal timing for cell therapy injection is essential to minimize side effects. Khailov et al. (54) used the Pontryagin maximum principle to study this problem, considering the presence of CAR-modified T-lymphocytes, B-leukemic or cancer cells, healthy B-cells, and inflammatory cytokines. The results suggest that therapies with resting intervals are more effective than continuous injections. Five possible scenarios were modeled, varying the injection time and analyzing the results based on the system parameters. According to their simulation, complete cancer recovery is achievable when chimeric cells preferentially target cancer cells over healthy ones.

**Table 1** provides a comprehensive overview of recent computational approaches and mathematical models on CAR-T cell therapy, categorized by scale (from protein to organism level), modeling approach, experimental context, and cancer type. Covering studies published between 2019 and 2024, it highlights various methodologies, including ordinary differential equations (ODEs), agent-based models, and machine learning approaches. Each study is linked to its relevant section in this review, illustrating how different modeling strategies address specific aspects of CAR-T therapy, from antigen receptor design to treatment optimization.

**Table 1:** Summary of computational approaches and mathematical models applied to CAR-T cell therapy (2019-2024).

Lead author	Year	Scale	Model/system	Experimental context	Parameter count	Simulation tool	Section	Cancer type	Tumor Type	PMID
Adhikarla V., et al. (18)	2021	organism	ODE	*in vivo*	9	Matlab	combination therapy	myeloma	liquid	34680320
Barros L. R. C., et al. (12)	2020	tissue	ODE	*in silico*	9	C	CAR-T cell expansion	leukemia	liquid	–
Barros L. R. C., et al. (39)	2021	cell	ODE	*in silico*	9	R	predator-prey	haematological	liquid	34208323
Brummer A. B., et al. (30)	2022	cell	ODE	*in vitro*	8	Python	cell dynamics	glioblastoma	solid	35081104
Brummer A. B., et al. (37)	2023	cell	ODE	*in vitro*	8	Python	predator-prey	glioblastoma	solid	37256133
Charoenkwan P., et al. (55)	2020	protein	RF and SVM	*in silico*	–	Python	antigen receptors	–	–	32333902
Cho J. H., et al. (56)	2021	tissue	boolean	*in vivo*	–	–	treatment efficiency	leukemia	liquid	33542232
Colina A. S., et al. (13)	2024	multi-scale	review	*in silico/in vitro*	–	Multiple	Manufacturing optimization	multiple	Multiple	39086435
Daniels K. G., et al. (57)	2022	protein	CNN and TSTM	*in vivo*	–	Python	treatment efficiency	leukemia	liquid	36480602
de Jesus Rodrigues, et al. (41)	2019	cell	ODE	*in silico*	10	Matlab	tumor response	lymphoma	liquid	–
Fischel H., et al. (20)	2021	tissue	agent based	*in silico*	7	Matlab	antigen receptors	breast	Solid	36231127
Giordano-Attianese G., et al. (58)	2020	protein	simulation	*in vitro | in vivo*	–	XML	treatment efficiency	–	–	32015549
Khailov E., et al. (54)	2020	tissue	ODE	*in silico*	15	Matlab	CRS	leukemia	liquid	–
Kimmel G. J., et al. (35)	2019	cell	ODE/SDE	*in silico*	9	Julia	CAR-T cell expansion	lymphoma	liquid	–
Kimmel G. J., et al. (32)	2021	cell	ODE/SDE	*in silico*	9	Julia	CAR-T cell expansion	lymphoma	liquid	33757357
León-Triana O., et al. (59)	2021	tissue	ODE	*in silico*	8	Matlab	time and dosage	leukemia	liquid	–
León-Triana O., et al. (50)	2021	tissue	ODE	*in silico*	11	Matlab	off tumor	glioblastoma	solid	33572301
Levin A. G., et al. (15)	2020	organism	ODE	*in vivo*	6	Matlab	time and dosage	breast	solid	32130452
Liu C., et al. (29)	2021	cell	ODE	*in silico*	6	–	cell dynamics	any	Any	33002189
Liu L., et al. (17)	2022	tissue	ODE	*in silico*	15	Monolixsuite	relapse	leukemia	liquid	36600553
Mahasa *K. J.*, et al. (19)	2022	cell	ODE	*in silico*	15	Matlab	combination therapy	–	solid	35430822
Martínez-Rubio A., et al. (46)	2021	tissue	ODE	*in silico*	18	Matlab	relapse	leukemia	liquid	34198713
Naghizadeh A., et al. (22)	2022	cell	CNN	*in vitro*	–	Python	antigen receptors	leukemia	liquid	35303007
Ottensen J. T., et al. (60)	2021	tissue	ODE	*in silico*	7	Matlab	time and dosage	–	–	34359690
Owens, K., et al. (61)	2021	tissue	ODE	*in silico*	19	Matlab	combination therapy	lymphoma, leukemia and melanoma	liquid	33740142
Paixão E. A., et al. (28)	2022	cell	ODE	*in silico*	19	C	cell dynamics	leukemia and lymphoma	liquid	36428671
Pérez-García V. M., et al. (45)	2021	tissue	ODE	*in silico*	7	Matlab	relapse	leukemia	liquid	39191245
Sahoo P., et al. (38)	2020	cell	ODE	*in vitro*	5	Matlab	predator-prey	glioma	solid	31937234
Santurio D. S., et al. (48)	2022	tissue	ODE	*in silico*	12	C	off tumor	glioblastoma	solid	–
Shah V., et al. (24)	2023	cell	Boolean/Logic	*in silico*	59	Matlab	downstream signaling	any	any	38083755
Stein A. M., et al. (53)	2019	molecule	ODE	*in silico*	5	Monolixsuite | R | Matlab	CRS	leukemia	liquid	30848084
Swanson E. R., et al. (44)	2022	tissue	ODE	*in vitro*	20	Matlab	tumor response	cancer stem cells	solid	38199607
Valle P. A., et al. (14)	2021	tissue	ODE	*in silico*	6	Matlab	time and dosage	leukemia	liquid	–
Zhang Z., et al. (16)	2022	tissue	ODE	*in silico*	14	Matlab	CRS	leukemia	liquid	36590643

The table categorizes studies by their modeling scale, approach, and application. The ‘Section’ indicates where each study is discussed in detail within this review. ODE, ordinary differential equation; SDE, Stochastic Differential equation; CNN, Convolutional Neural Network; TSTM, Time-Series Transformer Model; RF, Random Forest; SVM, Support Vector Machine.

## 4 Modeling in improving CAR-T cell therapy

### 4.1 Providing guidance for therapeutic strategies

Although CAR-T therapy has achieved great success in hematological malignancies, several challenges persist, including limiting adverse events, achieving complete and durable responses, and expanding the treatment to patients with solid tumors. Clinical studies have shown that higher dosages or multiple CAR-T cell infusions can potentially improve therapeutic outcomes in some patients with hematological malignancies, but this approach raises concerns about associated toxicity (62, 63). Additionally, the efficacy of CAR-T cell therapy in solid tumors are limited by factors such as tumor antigen heterogeneity, increased difficulty of T cell infiltration, and immunosuppressive tumor microenvironment. A promising strategy involves combining CAR-T cell infusion with other anti-tumor therapies, including chemotherapy, radiotherapy, oncolytic viruses (OVs), cancer vaccines, cytokines, and checkpoint blockade (64, 65). However, verifying therapeutic efficacy and safety through clinical trials is often resource intensive. Computational approaches offer a valuable tool by providing a systematic understanding of therapeutic responses based on complex treatment strategies.

### 4.2 Time and dosage

Control theory approaches offer advantages in managing the time and dosage of CAR-T cell therapies. Levin and colleagues (15) investigated how altering therapeutic concentration would affect tumor growth, observing high variability in the results. Increasing CAR-T cell number does not necessarily increase the killing ratio, as each CAR-T cell can kill multiple tumor cells. To address this dosing problem, Valle et al. (14) employed nonlinear systems theories like the Localization of Compact Invariant Sets (LCIS) and Lyapunov’s Direct method. LCIS identifies stable regions where system trajectories remain bounded over time, while Lyapunov’s method analyzes stability without requiring explicit solutions to equations by examining energy-like functions. Together, these approaches enable rigorous analysis of complex biological systems like CAR-T and tumor cell interactions. Their study presented four main dose delivery approaches: a constant daily dose, a constant weekly dose, or two doses at different periods and intensities. Using a protocol designed by Lee et al. (96), the authors found that the data achieved a complete response with CAR-T cells below the detectability threshold, outperforming other approaches. However, they noted discrepancies between their simulations and Lee et al.’s findings, which were more consistent with the biweekly administration schedule. The authors considered parameters like the cancer cell growth rate and killing efficacy rate of CAR-T cells, which they found to be highly patient- dependent. More comprehensive models of cell interactions can enable greater accuracy in understanding optimal therapy timing. León-Triana and colleagues (50) proposed a holistic approach to study cellular interactions during CAR-T therapies, including less commonly considered cells like hematopoietic stem cells. Their work demonstrated that varying time intervals for CAR-T therapies can be more advantageous in preventing cancer recurrence.

The quantity of CAR-T cells may also depend on several simultaneous complications. Ottesen and colleagues (60) investigated how external pathogens such as viruses and bacteria along with health complications such as aging, diabetes, and smoking, may influence cancer. The immune system may have limited capability, and external pathogens may compromise it, leading to cancer progression. The presence of CAR-T cells in the model can enhance the system’s ability to manage different external complications, as pathogens are considered. The model exhibits a bistable equilibrium with a dormant state and a cancer-infection disease state. The simulation demonstrated that cancer progression might depend on the treatment levels, and small perturbations can cause the system to transition from a low tumor concentration to a high tumor concentration, but not vice versa. One limitation of the work is the lack of specific infection to illustrate common scenarios. The authors primarily aimed to describe the potential limitations of the immune system in managing multiple complications. However, given the hypothesis of a limited immune system capacity, different pathogens would perturb it differently, demonstrating that not all external attacks would have the same effect.

These computational approaches also generate testable hypotheses about optimal dosing regimens that can be validated through clinical trials. For example, models predicting that biweekly administration achieves superior outcomes provide specific hypotheses for clinical testing, creating an iterative cycle where computational predictions guide trial design and clinical outcomes refine dosing models.

### 4.3 Combination therapy

Combination therapy, which involves using multiple therapies together for a beneficial outcome, has shown promise in reducing tumor growth with CAR-T cells, even if complete eradication is not achieved (18, 60). Combining CAR-T cells with OV, chemotherapy, radiotherapy, and immune checkpoint inhibitors is a common practice. OV therapies target selectively cancer cells, potentially enhancing the effectiveness of CAR-T therapies. Mahasa et al. (19) developed a model to investigate the effects of combining CAR-T cells with OVs on tumor cells. The model consisted of four ODEs that examined the infection rate, burst size, and viral clearance in the TME, and CAR-T cell migration. By expressing these biological processes as rates of change over time, ODEs provide a mathematical framework to simulate how these interconnected elements evolve together in response to combination therapy. The study also explored virus-induced increased levels in CAR-T cells’killing ability of tumor cells and performed simulations with and without considering virus-induced synergistic effects. Other experimental studies have shown that combining OVs and CAR-T cells can modestly decrease tumor growth, and understanding the potential synergistic effect and the time of administration is crucial (66, 67). Moreover, understanding the potential relationship between the OV and CAR-T cells may be important for future work. The findings suggest that combining OVs and CAR-T cells to treat tumor burden may not always result in better therapeutic outcomes, as CAR-T cells can have both beneficial and harmful effects.

**Figure 5:**
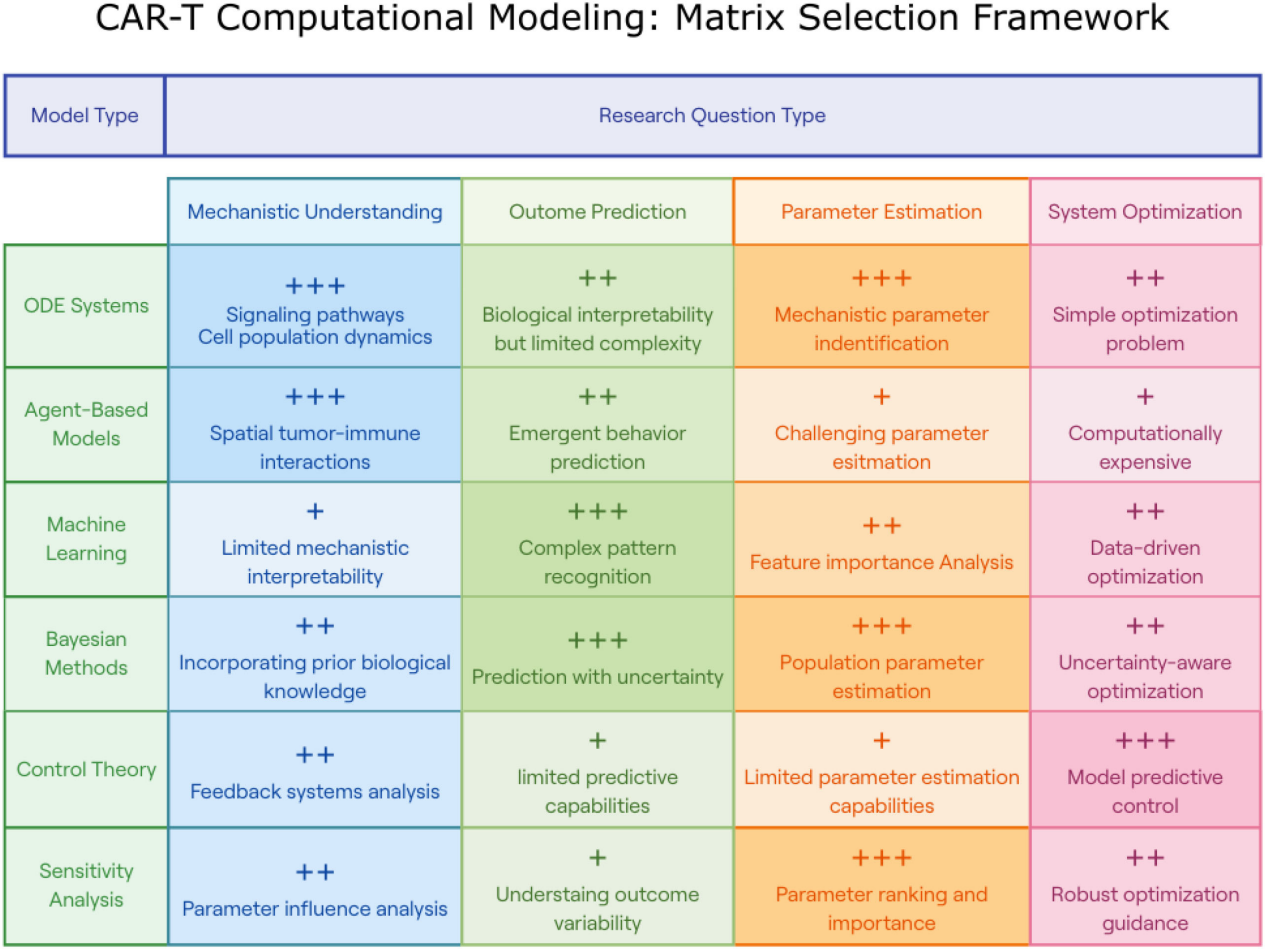
CAR-T Computational modeling: matrix selection framework. This figure presents a decision matrix for selecting appropriate computational modeling approaches based on specific CAR-T research objectives. The matrix categorizes six modeling methodologies (rows: ODE Systems, Agent-Based Models, Machine Learning, Bayesian Methods, Control Theory, and Sensitivity Analysis) against four research question types (columns: Mechanistic Understanding, Outcome Prediction, Parameter Estimation, and System Optimization). Each intersection is rated with plus signs (+, ++, or +++) indicating relative effectiveness and includes concise descriptions of strengths or limitations. This framework guides researchers in selecting optimal modeling approaches for their specific CAR-T research questions, highlighting that ODE systems excel in signaling pathway analysis, agent-based models in spatial tumor-immune interactions, machine learning in pattern recognition, Bayesian methods in uncertainty quantification, control theory in optimization, and sensitivity analysis in parameter ranking.

In addition, it is crucial to consider that a single dose may not be sufficient to eradicate tumor cells. Adhikarla and colleagues (18) investigated how the mathematical model for targeted radionuclide therapy (TRT) can be used to elucidate the dosing regimen, temporal scheduling, and sequential order of therapeutic interventions. The model focused on progression-free survival (PFS), overall survival (OS), and time to nadir. Administering CAR-T cell therapies before other treatments led to an increase in PFS, OS, and time to tumor burden nadir. Depending on the time between the two therapies, adapting the therapy based on the tumor’s proliferation rate, is essential. Both approaches emphasize patient preconditioning, which aims to create an environment where CAR-T cells can thrive and effectively target cancer cells. Owens and Bozic (61) investigated whether a mathematical model could be developed based on previous research in the field (68). They extended the work by modeling the population of CAR-T cells and the chemotherapy concentration. While the model is valuable for liquid tumors, its usefulness may be limited in solid tumors where the distribution of CAR-T and cancer cells is not uniform. Moreover, CAR-T resistance is still not explored with these methods. Through this work, they described a tumor-free equilibrium point and a high-tumor equilibrium. Moreover, understanding the potential relationship between the OV and CAR-T cells may be important for future work. **Figure 5** summarizes a decision matrix that guides researchers in selecting appropriate computational modeling approaches based on their research objectives, providing an overview of the strengths and limitations of various methodologies across mechanistic, predictive, and optimization tasks. To further clarify the distinctions between available computational strategies, **Table 2** provides a concise overview of major modeling approaches used in CAR-T cell research. It describes each method’s underlying principles, classification type (e.g., mechanistic vs. empirical, deterministic vs. stochastic), and the types of research questions it is best suited to address.

**Table 2:** Summary of mathematical modeling approaches for biological systems (2019-2024).

Modeling approach	Description	Type	Best used for
Ordinary Differential Equations (ODEs)	Mathematical equations that relate functions to their derivatives, describing how quantities change over time continuously and deterministically.	Deterministic, Mechanistic	Population dynamics, cell-cell interactions, cytokine signaling, when using average behaviors and large populations.
Stochastic Differential Equations (SDEs)	Differential equations containing random terms, accounting for inherent noise and variability in biological systems.	Stochastic, Mechanistic	Small cell populations, extinction events, cellular heterogeneity, when randomness significantly impacts outcomes.
Agent-Based Models	Computational models simulating actions and interactions of autonomous agents (cells), capturing emergent behaviors from individual rules.	Stochastic, Mechanistic	Spatial dynamics, cellular heterogeneity, cell-cell interactions in complex microenvironments, particularly in solid tumors.
Machine Learning Approaches (CNN, RF, SVM, TSTM)	Data-driven computational methods that learn patterns from existing datasets to make predictions or classifications.	Empirical, Statistical	Pattern recognition in large datasets, biomarker identification, predicting treatment outcomes from patient data, image analysis.
Pontryagin Maximum Principle	A mathematical approach for optimizing control systems, determining the best control strategy to minimize a cost function.	Deterministic, Optimization-based	Optimizing treatment protocols, dosing strategies, and timing of therapeutic interventions.
Markov Models	Stochastic models describing sequences of events where probabilities depend only on the current state, not previous history.	Stochastic, Statistical	Cell state transitions, modeling disease progression, predicting long-term outcomes from limited clinical data.
Boolean Networks	Models where variables take binary values (0/1) representing the state of a system component, with logical rules determining interactions.	Deterministic or Stochastic, Mechanistic	Signaling pathways, gene regulatory networks, designing logic-gated CAR systems.

The table categorizes computational techniques by their mathematical description, type, and application. The ‘Best Used For’ column indicates specific research contexts where each approach provides optimal insights.

Computational models of combination therapy serve as hypothesis-generating platforms, predicting synergistic effects and optimal sequencing that guide experimental design. These predictions can be systematically tested in preclinical models, with experimental results feeding back to refine combination therapy models and generate new hypotheses about therapeutic combinations.

### 4.4 Optimize CAR-T cell design

Optimizing chimeric antigen receptors (CARs) is a complex yet essential process. One promising new technique for this is Deep Mutational Scanning (DMS), which enables researchers to systematically introduce mutations across a CAR protein’s sequence, allowing for a detailed exploration of its functional landscape. Initially developed for simpler organisms like *E. coli* and yeast, DMS has recently expanded to mammalian cell systems due to technological advances, making it particularly valuable for studying proteins involved in human diseases. For instance, Di Roberto et al. (69) established a functional screening platform aimed at enhancing the selectivity and safety of CARs by integrating DMS with CRISPR-Cas9 genome editing techniques. This innovative approach enables the generation of a diverse library of CAR variants, which can then undergo high-throughput functional screening based on their ability to activate T cells in response to specific antigen levels.

Furthermore, CAR optimization strategies are increasingly incorporating machine learning (ML) approaches, such as variational autoencoders, as demonstrated by Yu et al. (70), alongside traditional mathematical models that serve as transparent ‘white-box’ tools. An example of this is PASCAR, a multiscale framework developed by Rajakaruna et al., which explores the design space for both constitutive and inducible CAR-T cells (71). In such optimization scenarios, researchers often encounter a Pareto front, where improving one parameter can inadvertently compromise another. Qiu et al. (72) addressed this challenge with CAR-Toner, a tool designed to balance effective tonic signaling without inducing CAR-T cell exhaustion or reducing persistence. Their approach refines positively charged patches (PCPs) on CAR surfaces to enhance CAR-T cell therapy performance. By integrating protein databases, structural biology, and advanced deep learning models, CAR-Toner efficiently calculates and optimizes PCP scores to improve therapeutic outcomes.

### 4.5 Enhance CAR-T cell signaling

Optimizing CAR-T cell signaling domains is crucial for improving therapeutic efficacy. Daniels and colleagues (57) aimed to systematically investigate CAR-T therapy design by using 13 signaling motifs (including a spacer motif) to create a repertoire of CAR costimulatory domains with random motif combinations. The goal was to study and identify specific combinations that could enhance stemness and cytotoxicity. As an example of their work, they predicted the effects of adding the M1 motif to CD28-like and 4-1BB–like synthetic costimulatory domains. While CD28-like domains were not predicted to enhance cytotoxicity or stemness, 4-1BB–like domains were forecasted to increase them. These predictions were experimentally validated.

Engineering cells may not be the only factor influencing treatment success or failure. Indeed, Giorgadze et al. considered different antigen distribution models defined as heterogeneous and binary antigen distribution models. In the first case, a gradient of distributions is presented. In the second case, there would be high or low levels of antigens without intermediate values. In both cases, the treatment was able to eliminate the tumor by targeting cancer stem cells in addition to the bulk of the tumor (73).

Recent advancements in CAR specificity include the work of Ruffo et al., who developed a novel approach for enhancing receptor specificity and control in CAR and synthetic Notch (synNotch) therapies (74). This method uses a “universal” receptor system with covalent antibody attachment, allowing post-translational modification of targeting. A SNAPtag fused to the receptor enables it to bind covalently with antibodies carrying a benzyl guanine (BG) motif. This system’s benefits include high-affinity interactions essential for receptor activation and the ability for a single receptor to target multiple antigens using different BG-tagged antibodies.

### 4.6 Improve CAR-T cell specificity

Treatment specificity can be enhanced by designing new proteins. Giordano-Attianese et al. (58) studied the structure of proteins using Rosetta MotifGraft (75), introducing a “safety switch” that allows for the modulation of T cell effector function with a small molecule drug adding a controllable element to synthetic cellular therapies. Tuning down CAR-T cells can offer potential benefits, such as reducing the cost of recombinant protein production and potential immunogenicity. Additionally, a safety switch allows for faster and more precise control over cell therapies.

Designing new chimeric antigen receptors can also be achieved through Boolean logic. Current efforts are focused on developing a three-input logic system to identify specific antigens, enabling biocomputation in human immune cells. Biocomputation, along with CAR design, is possible in both CAR-T cells or other cell types. In the work of Hwan Cho et al. (56), a SUPRA CAR system, that uses boolean logic, has been introduced on seven types of cells. This system has the potential to induce helper T cell activation at a specific time, creating a sequential order of inflammation and reducing cytokine release syndrome without compromising the initial anti-tumor response. Future research in this area is likely to expand the range of antigens that are not solely cancer specific.

T cell antigen receptors (TCRs) are transmembrane proteins expressed on T cells that recognize specific antigens, typically foreign molecules that elicit an immune response. Chimeric antigen receptors (CARs), engineered to recognize distinct antigens, are another avenue of focus. Charoenkwan et al. (55) developed an open-access platform utilizing machine learning algorithms, such as support vector machines (SVM) and random forests (RF), to identify T cell epitopes in tumor antigens. Leveraging multiple features, this approach improved performance by 4% to 7% over previous works, significantly reducing the time and cost of experimental T cell epitope identification in tumor antigens. To address data imbalances, the authors oversampled sparse data, enhancing the accuracy of their results.

## 5 Future directions

### 5.1 Goal of modeling

**Figure 6:**
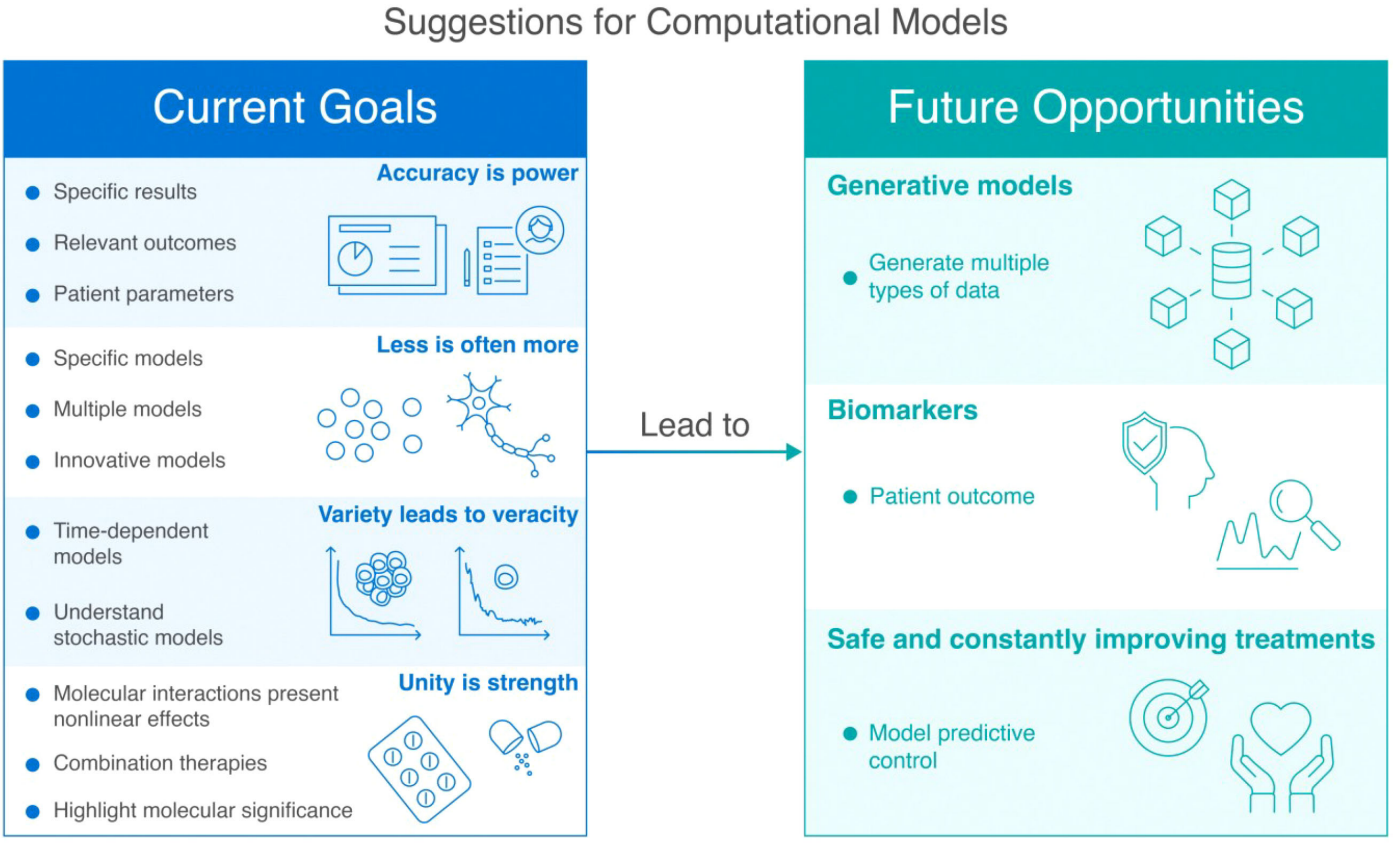
Comprehensive framework showing the evolution of computational modeling for CAR-T cell therapy. The left panel outlines current research objectives organized under four foundational principles: (1) ‘Accuracy is power’ emphasizes the importance of patient-specific parameters and clinically relevant outcomes in models; (2) ‘Less is often more’ advocates for focused modeling approaches that prioritize essential interactions over comprehensive but unwieldy systems; (3) ‘Variety leads to veracity’ highlights how diverse modeling techniques (from deterministic to stochastic approaches) provide complementary insights into complex biological processes; and (4) ‘Unity is strength’ underscores the value of integrating multiple therapeutic approaches and recognizing nonlinear effects in combination therapies. The right panel illustrates how these current approaches are evolving toward three transformative future opportunities: generative models that can synthesize and analyze multiple data types simultaneously; biomarker development for precise prediction of patient-specific therapy responses and toxicities; and safe, continuously improving treatment strategies through predictive control methods. The connecting arrow represents the natural progression from current modeling goals to more advanced computational approaches, demonstrating how today’s foundational principles will enable tomorrow’s precision medicine applications in CAR-T therapy.

The objective of this study was to elucidate the computational approaches and mathematical models pertaining to CAR-T therapeutics. As illustrated in **Figure 6**, current modeling approaches follow key principles ranging from accuracy to unity, while future opportunities lie in advanced computational techniques and improved patient outcome prediction. Although the field of synthetic biology predominantly focuses on the engineering of CAR-T cell components, this review underscores the importance of pharmaceutical products that consider crucial factors such as patient cost, time, dosage, and age. In summary, the following key points must be kept in mind:

#### 5.1.1 Accuracy is power

In order to determine precise and relevant outcomes, it is imperative to tailor each model to a specific patient or population. Mathematical models must consider important factors such as the chimeric antigen receptor generation (45, 58), patient age, other concurrent treatments (30), and the variability in cellular gene expression (17). Model fitting for individual patients may rely on specific parameters obtained through specialized tests (12, 38) and, the nature of the test and its capacity to measure certain parameters should also be considered. For example, bone marrow presents challenges in studying certain features that may be restricted to hard-to-reach sites (46).

#### 5.1.2 Less is often more

Previous papers have considered various components such as CAR-T cells, cancer cells, memory cells (39), neurons (48), and stem cells (50), among others (55, 61). However, no single model has described all of them. While holistic models can provide a general understanding of global behavior, they are often computationally intensive and may not yield accurate predictions for patients. Therefore, identifying and studying specific interactions is crucial. Mathematical equations can lead to significant progress, but a biological foundation is essential to remain connected to real-world issues, such as cellular dynamics (between logistics or Allee effect) and dual CAR-T therapy (37). A conservative approach can help acquire information about the system being studied and formulate hypotheses, such as dual CAR-T therapy or system evolution (28, 29). Imaging techniques will play a pivotal role in the coming years (22, 41, 55), where obtaining less data is preferable to obtaining erroneous data for CAR-T cell design.

#### 5.1.3 Variety entails veracity

The use of ODEs is a prevalent approach for studying cellular interactions due to their computational efficiency and mechanistic nature. However, developing more comprehensive models that represent various stages of CAR-T cell development, such as cancer treatments, is essential. Multiple differential equations can describe cell distribution, expansion, contraction, and persistence at specific time points (14, 53). Additionally, ODEs may not function effectively with small sample sizes, and SDEs may be more advantageous in such cases. Examples can be found in the works of Kimmel et al. (32, 35), where stochasticity better represented a low number of cells. Therefore, various modeling techniques can enhance veracity by providing a more comprehensive view of the cellular interactions involved in CAR-T cell development.

#### 5.1.4 Unity is strength

In scientific research, understanding the interconnectedness of systems is crucial. While therapies often focus on single components, the nonlinear effects of interconnectivity can make a single therapy insufficient. Mathematical models should consider how multiple therapies could work together to achieve a beneficial effect in cancer treatments, while minimizing collateral damage such as CRS (18, 19, 61). Efforts should be made to unite cell therapies with molecules, as they present distinct characteristics that must be considered (16, 44, 53). While CAR-T cells are known for their remarkable qualities like increased size after injection, prolonged persistence in the body, and high specificity, molecules may offer advantages due to our prior knowledge of pharmacokinetics, rapid clearance, and diverse actions.

### 5.2 Future opportunities

As a powerful tool that frees experimentalists from exhaustive testing conditions, computational approaches and mathematical models of CAR-T therapeutics will continue to evolve in the following directions:

#### 5.2.1 Enhanced modeling assisted by advanced experimental results

Detailed experimental data can be used to develop accurate predictive models for CAR-T cells with more advanced designs and to refine model parameters. For example, modeling has been used to characterize the signaling event of the CAR-T cell design with either CD28 or 4-1BB costimulatory domains. However, there are limited modeling studies describing the signaling response of CD28 and 4-1BB CAR-T cells, which have been extensively studied in preclinical research and early-phase clinical trials.

Additionally, computational approaches should increasingly guide the rational engineering of CAR constructs themselves, optimizing binding kinetics, signaling domains, and logic-gated designs to improve therapeutic performance before experimental validation.

Furthermore, there is a growing need to develop models describing the activities of fourth and fifth generation CAR-T cells, which have gained increasing interest in recent years. To address the challenges in solid tumor treatment, additional variants of logically gated CAR-T cells or spatially and temporally controllable CAR-T cells, have been developed such as dual CARs, split CARs and inducible-split CARs, small molecule-inducible, optogenetic and sonogenetic CAR, and suicide CAR-T cells. The increased complexity of these CAR designs exponentially increases the workload required for characterizing and evaluating them through traditional experimental methods. Computational approaches and mathematical models can significantly accelerate these studies, although challenges remain in establishing them.

The activation kinetics, cytokine signaling pathway and cytokine secretion are regulated by CAR activation and additional conditions, leading to increased complexity in both their cellular signaling response and *in vivo* dynamics, proliferation, and killing. Using more detailed experimentally determined parameters as input values can greatly aid in forming and constraining the models. Various non-invasive imaging techniques can be used to monitor CAR-T cellular and molecular events, including *in vitro* fluorescent proteins (FPs) and biosensors-based visualization; *in vivo* fluorescence, and bioluminescence imaging, such as Magnetic Resonance Imaging (MRI), Positron Emission Tomography (PET), and photoacoustic (PA) imaging. These imaging techniques provide comprehensive understanding of CAR-T cell signaling pathways and real-time biodistribution and activation. The results can serve as input parameters for future modeling work, leading to the mutual development of experimental and computational/mathematical studies.

#### 5.2.2 Integrated modeling and other computational techniques for CAR-T cell design

Combined with other *in silico* approaches, modeling can be leveraged to guide the design of CAR extracellular binding epitopes and intracellular signaling motifs. In the past, CAR structural design was dominated by rational design, library screening, and experimental verification. Recently, computational approaches, such as molecular dynamic, molecular docking, and AI-based protein discovery, have shown great promise in CAR molecule design by significantly expediting the design process and reducing cost and workload. However, numerical approaches are not a full substitute for experimental approaches. Experimental confirmation remains crucial to ensure the accuracy and efficacy of computational predictions, while modeling can be employed to guide the design of these validation experiments. Therefore, the integration of modeling with other computational techniques offers a more effective way to accelerate CAR-T cell design.

To achieve a balance between anti-tumor activity and the risk of toxicity or other side effects in solid tumors, a research team computationally designed a chemically disruptable heterodimer (CDH). In this design, the antigen-recognition chain and the CD3ζ- and CD28-containing endodomain signaling chain were placed on separate subunits. A small-molecule drug was administered to block the binding between the two chains, serving as a safety switch by dynamically inactivating CAR-T cell activity. Tonic signaling, the spontaneous activation of CAR-T cells in the absence of tumor antigen stimulation, plays a crucial role in controlling CAR-T efficacy. In another study, researchers numerically determined the 3D conformation, electrostatic potential, surface electrostatic and net charge of CAR scFvs. By combining these computational results with experimental approaches, they concluded that positively charged patches (PCPs) on the surface of the CAR antigen-binding domain mediate CAR clustering and result in tonic signaling. Fine-tuning these PCPs can optimize tonic signaling and CAR-T cell fitness. Molecular docking was also employed to optimize the anti-tumor effect of anti-CD123 CAR-T cell against acute myeloid leukemia (AML). A research team investigated the interaction between anti-CD123 and CD123 complex using molecular docking, proposing mutations in anti-CD123 antigen binding loops to refine the design. Together with experimental validation, they were able to adjust CAR expression and CAR binding affinity without altering the overall CAR design.

#### 5.2.3 Modeling in the era of artificial intelligence

The field of CAR-T cell therapies stands to be significantly impacted by computational techniques and methodologies that have shown promise in other fields and now hold transformative potential here.

##### 5.2.3.1 Large language models

Large language models (LLMs), which are AI systems trained on vast amounts of text, offer a powerful means of consolidating and synthesizing the extensive, often fragmented body of biological knowledge. In biology and medical research, information is scattered across numerous publications, each adding unique perspectives, findings, and methodologies that can be challenging to integrate due to the sheer volume of literature. This challenge is particularly pronounced in the CAR-T therapy field, where rapid advances in tumor antigen discovery, immune response modulation, and cellular engineering have generated a highly dynamic research landscape.

LLMs could reshape CAR-T research by serving as comprehensive, adaptive tools capable of summarizing key findings, identifying emerging trends, and even detecting subtle patterns that might elude individual researchers. By synthesizing insights across related topics—such as antigen identification, receptor engineering, safety mechanisms, and toxicity management—LLMs can provide a cohesive perspective that highlights previously unrecognized therapeutic opportunities or challenges. Additionally, LLMs can analyze complex interconnections between studies, potentially aiding in hypothesis generation for CAR design optimization or reducing off-target effects. These capabilities also extend to identifying research gaps, informing experimental design, and enhancing translational potential by highlighting aspects crucial to clinical applications.

For example, Chaves et al. developed a model fine-tuned for therapeutic development tasks across diverse modalities, including small molecules, proteins, nucleic acids, and cell lines (76). This model leverages the Therapeutics Data Commons (TDC), an extensive dataset, to achieve competitive performance on multiple benchmarks in areas like drug-target binding, toxicity prediction, and drug combination synergy. Such projects can predict interactions involving cell lines and other biological entities. Another example comes from Li et al., who developed a model specifically fine-tuned for predicting drug synergies in rare tissue types (77). This model addresses challenges in synergy prediction for underrepresented tissues in cancer research, where limited experimental data and structured information are available.

##### 5.2.3.2 Generative AI beyond text

While large language models (LLMs) are transforming the synthesis of text-based knowledge, other generative AI techniques are expanding possibilities beyond text alone, working across various data types such as images, molecular structures, and even genomic sequences. These broader generative AI models offer transformative potential in fields like genomics, cellular therapies, and drug development, where data and discoveries are scattered across studies with diverse methodologies. By simulating biological systems, proposing hypotheses, and designing novel molecules or genetic sequences, these models can accelerate research in ways that LLMs alone cannot.

Some studies have started to explore the unique advantages of these broader generative AI approaches in biomedicine (78, 79). Current applications frequently focus on designing and predicting interactions for small molecules (80–82), though future research may apply similar techniques to larger molecules (83) and even cell therapies, opening up new avenues for personalized and regenerative medicine. Beyond their current applications, generative AI models could reshape biomedical research by enabling more holistic approaches to understanding and intervening in complex diseases. For example, by integrating multi-omic data (genomic, transcriptomic, proteomic) with clinical and environmental data, these models could help identify patterns that reveal underlying disease mechanisms or patient-specific therapeutic targets. As research pushes towards greater personalization in medicine, these AI models could support the design of tailored treatments, including custom-engineered cell therapies. The long-term potential of these models lies not only in drug discovery but in bridging the gap between data and clinical application, bringing precision medicine closer to becoming a standard of care.

##### 5.2.3.3 Model predictive control

In addition to generative AI, other advanced computational techniques are proving valuable in optimizing CAR-T cell therapies. Model Predictive Control (MPC) (84–86) is an advanced control strategy widely used in engineering and process systems. It involves utilizing a model of the system to predict its future behavior and optimize control inputs in real time, all while considering constraints on the system’s states and control actions. In MPC, the control action is determined by solving an optimization problem at each time step, with the objective of minimizing a cost function related to system performance, subject to various constraints.

The advantage of MPC over other techniques lies in its ability to optimize performance while managing constraints. In the context of CAR-T cell therapies, this approach is particularly beneficial as it enables the definition of constraints across multiple stages, from production and distribution to delivery and injection. By effectively managing these constraints, MPC can enhance the efficiency, consistency, and quality of cell-based therapies.

Looking ahead, MPC is poised to make a significant impact on the medical field, especially as it becomes applicable to increasingly complex processes. A key benefit of MPC is its ability to operate with relatively limited data compared to many data-driven approaches, which typically require large datasets for model training. This makes MPC an appealing option in areas where data is scarce or expensive to collect (87). Moreover, integrating contextual knowledge—such as biological insights and system dynamics—becomes essential for applying MPC in healthcare, ensuring that predictions and optimizations are grounded in real-world conditions.

By focusing on models that combine predictive accuracy with contextual understanding, MPC can streamline the production and delivery of CAR-T cell therapies while offering substantial advancements in personalized medicine, ultimately improving the accessibility, cost-effectiveness, and safety of these treatments.

##### 5.2.3.4 Tracking treatment efficacy through biomarkers

Biomarkers are measurable indicators of biological processes, conditions, or diseases within the body, providing essential insights into an individual’s health status, disease progression, and response to treatments. They come in various forms, including digital biomarkers, which capture data through electronic devices, and biological biomarkers, which are measurable substances found in biological samples such as blood, urine, or tissues.

Digital biomarkers are increasingly valuable for real-time monitoring of patient responses. They can reveal patterns like changes in vital signs or early signs of adverse events, such as Cytokine Release Syndrome (CRS), a common complication in CAR-T cell therapies. This continuous data flow enables clinicians to swiftly adjust treatment plans, enhancing patient safety and ensuring precise therapeutic outcomes (88). For example, digital biomarkers (89) can help detect subtle shifts in a patient’s immune response or alert physicians to potential complications, enabling timely intervention (88).

Biological biomarkers, on the other hand, reflect specific biological processes or disease states. In the context of CAR-T cell therapies, biological biomarkers are crucial for evaluating therapy effectiveness and the behavior of engineered T cells in the patient’s body (90). By tracking tumor antigen levels, clinicians can assess whether CAR-T cells are effectively targeting and eliminating cancer cells. Additionally, immune cell markers help monitor whether CAR-T cells are expanding and persisting at necessary levels to sustain therapeutic efficacy or even a joint immunotherapeutic reaction (91, 92).

Aging biomarkers offer further insights into how immune decline or cellular aging may influence the success of CAR-T therapies. This is particularly relevant for older patients, whose immune systems may respond differently. Understanding these biomarkers allows for better therapy management and customization based on the individual’s age and immune status (93–95), ultimately improving the likelihood of successful outcomes in older populations.

##### 5.2.3.5 In conclusion

This review has provided a comprehensive analysis of mathematical models and computational approaches for CAR-T cell therapeutics, highlighting recent advances and emerging opportunities in this rapidly evolving field. The review demonstrates how computational tools can enhance our understanding of CAR-T cell behavior across multiple scales, from molecular interactions to clinical outcomes.

### 5.3 Summary of key findings

Our review has revealed several important trends in the computational modeling of CAR-T therapeutics:

- Receptor Design and Signaling Dynamics: Mathematical models have successfully characterized the impact of CAR designs, antigen expression, and downstream signaling events on treatment efficacy. In particular, studies by Finley et al. (10, 23), Reiter et al. (11), and Fischel et al. (20) have provided crucial insights into how CAR co-stimulatory domains influence cell activation and how antigen expression levels correlate with treatment efficacy.
- *In Vivo* Response Prediction: Computational approaches have advanced our ability to predict CAR-T cell dynamics, expansion, killing efficiency, and tumor responses. Models by Paixao et al. (28), Liu et al. (29), and Brummer et al. (37) have characterized important factors in CAR-T cell efficacy and identified thresholds for therapeutic success.
- Side Effects Management: Models addressing cytokine release syndrome and on-target/off-tumor effects have provided valuable frameworks for enhancing treatment safety. Zhang et al. (16) and Stein et al. (53) have shown how interventions like tocilizumab administration can mitigate CRS severity while maintaining therapeutic efficacy.
- Therapeutic Strategy Optimization: Computational approaches have provided insights into optimal dosing regimens, timing, and potential combination therapies. Work by Valle et al. (14), León-Triana et al. (50, 59), and Adhikarla et al. (18) has demonstrated how mathematical models can guide therapeutic decision-making.

## Glossary

CAR-T: Chimeric Antigen Receptor T-Cell
FDA: Food and Drug Administration
scFv: Single-Chain Variable Fragment
IL: Interleukin
STAT: Signal Transducer and Activator of Transcription
JAK: Janus Kinase
IS: Immunological Synapse
AICD: Activation-Induced Cell Death
TAA: Tumor-Associated Antigens
ALL: Acute Lymphoblastic Leukemia
CLL: Chronic Lymphocytic Leukemia
MM: Multiple Myeloma
CRS: Cytokine Release Syndrome
NK: Natural Killer (cell)
SJRs: Synthetic Juxtacrine Receptors
TRUCKs: T Cell Redirected for Universal Cytokine-Mediated Killing
ODE: Ordinary Differential Equation
SDE: Stochastic Differential Equation
CNN: Convolutional Neural Network
TSTM: Time-Series Transformer Model
RF: Random Forest
SVM: Support Vector Machine
AML: Acute Myeloid Leukemia
PET: Positron Emission Tomography
MRI: Magnetic Resonance Imaging
PA: Photoacoustic (Imaging)
CDH: Chemically Disruptable Heterodimer
PCP: Positively Charged Patches
LLM: Large Language Model
TDC: Therapeutics Data Commons
MPC: Model Predictive Control
PFS: Progression-Free Survival
OS: Overall Survival
TRT: Targeted Radionuclide Therapy
CRISPR: Clustered Regularly Interspaced Short Palindromic Repeats
TCR: T Cell Antigen Receptor
synNotch: Synthetic Notch
BG: Benzyl Guanine
TME: Tumor Microenvironment
OT: OT, On-Target, Off-Tumor
BCR: B Cell Receptor
