# Correction: Mathematical models and computational approaches in CAR-T therapeutics

**DOI:** 10.3389/fimmu.2025.1694607

**Published:** 2025-09-08

**Authors:** Guido Putignano, Samuel Ruipérez-Campillo, Zhou Yuan, José Millet, Sara Guerrero-Aspizua

**Affiliations:** ^1^ bioERGOtech, Taranto, Italy; ^2^ Department of Biosystems Science and Engineering, ETH Zurich, Basel, Switzerland; ^3^ Department of Computer Science, ETH Zurich, Zurich, Switzerland; ^4^ AI Center, ETH Zurich, Zurich, Switzerland; ^5^ Alfred E. Mann Department of Biomedical Engineering, University of Southern California, Los Angeles, CA, United States; ^6^ ITACA Institute, Universitat Politecnica de Valencia, Valencia, Spain; ^7^ Universidad Carlos III de Madrid, Departamento de Bioingeniería, Centro de Investigación Biomédica en Red de Enfermedades Raras-ISCIII, Instituto de Investigación Sanitaria Fundación Jiménez Díaz, Centro de Investigaciones Energéticas, Medioambientales y Tecnológicas, Madrid, Spain; ^8^ Centre for Biomedical Research (CIBM), University of Granada, Granada, Spain

**Keywords:** synthetic biology, biological system modeling, CAR-T cells, mathematical modeling, computational immunotherapy, therapeutic optimization, T cell engineering

In the **Acknowledgements**, a final sentence is missing.

It should be:

“Guido Putignano acknowledges support from the Mercatus Center’s Emergent Ventures Fellowship and the Foresight Institute Fellowship Program during the development of this work. We thank Sonia Monti for designing the figures and Professor Alessandro Colombo from Politecnico di Milano for his initial support, conceptual guidance, and methodological feedback.”

The original version of this article has been updated.

